# Not just avoidance: dogs show subtle individual differences in reacting to human fear chemosignals

**DOI:** 10.3389/fvets.2025.1679991

**Published:** 2025-09-15

**Authors:** Svenja Capitain, Friederike Range, Sarah Marshall-Pescini

**Affiliations:** Domestication Lab, Department of Interdisciplinary Life Sciences, Konrad Lorenz Institute of Ethology, University of Veterinary Medicine Vienna, Vienna, Austria

**Keywords:** dogs, companion animal, chemosignals, fear, olfactory, dog-human interaction, emotional contagion

## Abstract

Recent olfactory studies suggest that human emotional chemosignals can alter dog behavior. However, their methods impeded a firm conclusion on whether dogs reacted to the scent directly or to the present human’s unconscious response to the intraspecific stimulus. Moreover, whether these reactions differ between dogs has not yet been explored. Therefore, we investigated dogs’ reactions to human fear or neutral chemosignals while shielding the present human from the smells. Dogs were first trained to approach a single empty target on command, before they were given the choice between two targets laced with human smell (experimental group (*n* = 41): one fear target and one neutral; control group (*n* = 20): both neutral targets). Dogs in the experimental group stayed longer with the experimenter, displayed lower tail posture, and took longer to approach a target than control dogs, though target choice did not differ at the group level. Age and sex showed no effect. Furthermore, dogs in the experimental group compared to the control group showed stronger interindividual variation in how quickly they approached one smell over the other and how many commands they required. This finding suggests that dogs are indeed influenced by human fear smell beyond the humans’ reaction, though it challenges previous assumptions of an innate interspecific fear avoidance. The influence of life experience or breed on the individual differences may be worth exploring to better understand and guide dogs’ experience of the world.

## 1 Introduction

Dogs have evolved closely with humans for millennia (1). They can discriminate between the emotional information in human facial expressions (2–5), vocalizations (6), and body language (7) and respond accordingly (5, 8, 9). While most studies focused on visual and auditory cues, recent research has explored dogs’ primary sense – smell – and the role of human chemosignals (10–12). Chemosignals are chemical substances that animals (including humans) excrete consciously or unconsciously to alter others’ behavior, including recognition, mating, and alarm signals (13, 14). Recent evidence suggests that emotions also have distinct chemosignal signatures, eliciting similar emotional and physiological states in intraspecific recipients [e.g., humans: (15, 16), dogs: (17)].

The consideration of dogs’ co-evolution and daily life with us humans has sparked investigations into our interspecific communication through these emotional chemosignals (11, 12, 18). Perhaps unsurprisingly, dogs can be trained to distinguish the smells of different human emotions (19, 20). Additionally, studies have evidenced that dogs exposed to human fear sweat samples spontaneously showed more owner-seeking and higher heart rates than with happiness samples (11, 21, 22). Similarly, dogs exposed to human fear in a Judgment Bias Task showed greater pessimism than dogs exposed to happy chemosignals (23). These results were hence interpreted as olfactory-based interspecific emotional contagion between dogs and humans (11, 12, 23).

However, in all these studies, a human was exposed to the scent alongside the dog during the test, either handling the sample (17, 23) or remaining in close proximity (11, 20–22). This is surprising, given ample evidence of how easily dogs react to a handler’s physiological modulations (24, 25) and subtle behaviors, including facial expressions (7, 26, 27). As mentioned above, the smell of human fear elicits unconscious reactions in other humans, both physiological (e.g., neural fight or flight activation) and behavioral (e.g., fearful facial expressions) (15, 28, 29). This simultaneous exposure of humans and dogs in these studies therefore impedes a firm conclusion on whether dogs react to the chemosignal itself or to human behavioral changes.

Additionally, these studies exposed dogs separately to different human emotional chemosignals, either in between-subjects designs (11, 21, 22) or different conditions (23), limiting investigations of potential variability in dogs’ reactions to human fear scent. Concordantly, the persistent owner-seeking behavior during fear-smell exposure across studies, which was already present in 6-month-old puppies, had been interpreted as an innate reaction of dogs to human fear chemosignals (22). However, a learned response or prey appraisal may be equally plausible (12). Supporting this finding, dogs use different nostrils for sniffing human vs. canine fear cues (17), suggesting distinct neural pathways rather than an automatic fear response. Given dogs’ diverse demography, life experience, and breed functions, we hypothesize that – assuming the dogs react to the smell itself – some dogs may develop an avoidance response to human fear, while others approach it.

Together, these methodological gaps may have biased our interpretation of dogs’ behavior toward human fear smell, impeding not only our understanding of how dogs experience their interactions with human emotions and the environment, but also the exploration of individualized mitigation strategies if dogs indeed react to the smell itself. Thus, the aim of the current study was two-fold. First, dogs’ reactions to human fear chemosignals were investigated, while preventing the present humans from reacting to the intraspecific chemosignals. Since human-directed behaviors were central in the previous chemosignal studies, we kept the human in the setup but shielded them from the smells’ influence through a mask and gum chewing (30, 31). Second, interindividual variability was tested when given a choice between human neutral and fear chemosignals. Hence, we adopted a between- and within-subject design, allowing subjects to manifest both approach and avoidance behaviors toward the fear and control scents. Dogs were first trained to touch an empty target on command, which served as a foundation for the choice task during testing. In the test, the dogs were presented with two targets laced either with human fear and neutral chemosignals (experimental group) or both with human neutral chemosignals (control group). Across ten trials, the animals were given the choice to accomplish the command at their preferred target. We analyzed approach and avoidance tendencies, human-directed behavior, and tail posture.

We hypothesized that if dogs’ negative reaction to the fear smell in the previous studies was independent of human influence, we would see more target avoidance, low tail postures, and perhaps human-directed behavior (given that the experimenter was familiar but not the owner) in the experimental vs. control group. Alternatively, or additionally, considering the possibility of individual differences, e.g., due to age, sex, different life experiences, or learned responses, we further expected that there may be interindividual variation in some of the behavioral reactions.

## 2 Methods

### 2.1 Ethical approval

The study received ethical approval from the ‘Ethik und Tierschutzkommission’ of the University of Veterinary Medicine Vienna for the dogs (Ref.: ETK-031/03/2024) and the Ethikkommission of the FH Campus Wien for the human scent donors (Ref.: 262/2025). All scent donors and dog owners gave written informed consent for their (dogs’) participation in the study and the use of the resulting data and video.

### 2.2 Scent collection

Sixteen female students (average age 25.3 years), unfamiliar with the dog participants, were sampled at the University of Veterinary Medicine, Vienna. Following standard protocols (11, 22, 23, 32), each donor watched a 23-min nature narration (neutral smell) and horror scenes (fear smell) (“The Passenger,” “Nighty Night, Nancy,” “Vicious,” “Mr Creak”; for details see Supplementary material) [scenes validated by (29, 33)] alone in a darkened room while wearing sterile absorbent compresses (Cutisorb, BSN Medical) in their axillaries. Participants were non-smokers, outside the fertile phase of their cycle, and avoided odorous foods and products for 16 h prior to sampling. Our within-study design necessitated that the two samples from each participant differ only in the emotion. Following Wilson et al. (20), each participant therefore first watched the neutral movie (“Smell 1”), wiped their arms after sample processing was completed and then repeated the same procedure with the second movie (“Smell 2”). Control group donors watched the neutral movie again for Smell 2, while experimental group donors watched the horror movie second. The movies’ effectiveness was confirmed using Spielberger’s State–Trait Anxiety Inventory (34, 35) pre-post exposure (see Supplementary material for analysis). Immediately after each movie, each absorbent was cut into four pieces, and the participant blew their breath on them (23). Samples were stored at −20 °C.

### 2.3 Subjects

Sixty-two pet dogs (≥1 year old, various breeds, Supplementary data) were recruited through the Clever Dog Lab Database (Messerli Institute, University of Veterinary Medicine, Vienna), social media, and a local dog school. One dog failed training, leaving 61 dogs to be randomly assigned to the experimental group [*n* = 41, 26 females, 15 males, mean age (SD) 5.9 years (2.6)] and control group [*n* = 20, 10 females, 10 males, mean age (SD) 5.85 years (3.9)].

### 2.4 Experimental setup

**Figure 1:**
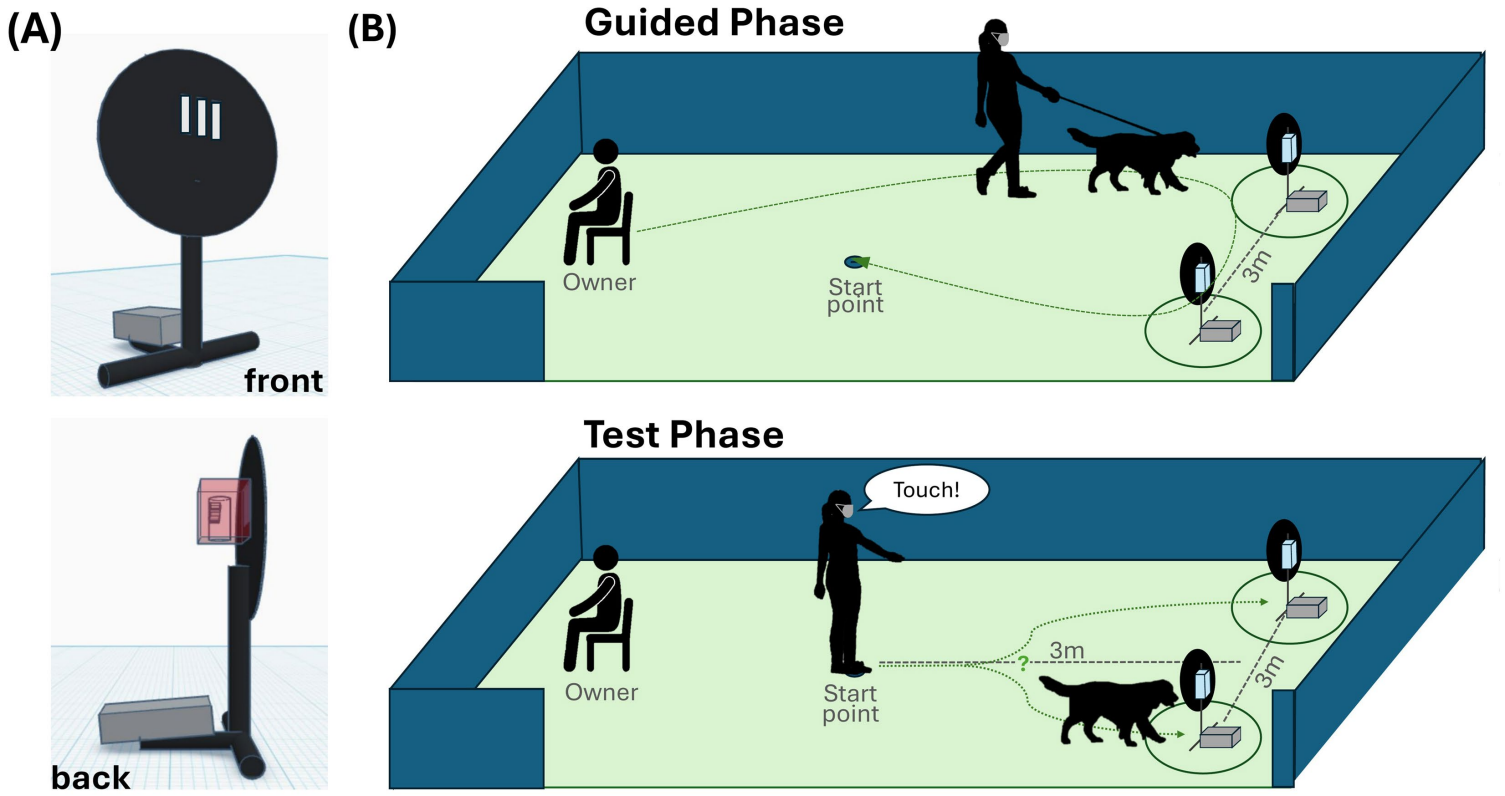
Setup. **(A)** The target from the front (top) and side (bottom), and **(B)** the test setup with the Guided Phase (top) and Test Phase (bottom), and the walking trajectory (green dotted line).

The study was conducted in a fenced outdoor area at the Clever Dog Lab (*n* = 53), the local dog school (*n* = 7), or the participants’ home (*n* = 1). A black plastic disc (d = 40 cm) on a short metal pole acted as the “target,” fixed in place with a cobblestone (Figure 1A). Three cutouts in the plastic disc connected to a plastic box at the back of the disc that held the sample tube. The study comprised one to four training sessions, followed by one test session. Each session was conducted on a different day.

### 2.5 Training procedure

Each session started with the dog freely exploring the area. The experimenter used positive reinforcement to train the dogs on a single target (empty, no scent) based on a combined hand and voice command (see Supplementary Video S1). The maximum length of a training session was 15 min of active training time, broken up by 5-min breaks according to the dogs’ engagement level. Since dogs rely less on their nose in more automated tasks (36, 37), training was aimed at getting dogs to reliably approach the target to a minimum of 20 cm proximity on command while keeping training minimal. Therefore, dogs were trained on their first offered movement: nose touch (*n* = 38), paw touch (*n* = 6), or running closely around the target (*n* = 17). This approach ensured an inclusive sample, with only one of the 61 dogs failing training. Dogs were considered trained once they successfully completed the command three times in a row from 3 m away (1–4 sessions, average 1.3), receiving a food reward each time after being called back to the experimenter. The test was conducted in the next session. The point of training the dogs for the approach was to facilitate analyzable choosing and avoidance behavior when exposed to the scents in the test.

### 2.6 Test procedure

Two sample tubes were defrosted 30-min before testing, either both neutral (control group) or one neutral tube and one fear tube (experimental group). Each tube contained sweat samples from two donors sampled in the same condition. Tubes were marked to track placement and pairings, but their content was concealed to keep the experimenter blind to group and smell allocation (for blinding procedure see Supplementary material).

The session started with three warm-up trials where the dog approached a single, centrally located, empty target on command for an experimenter-delivered food reward. The dog was then leashed and seated with the owner 6 m from the setup, facing away. The experimenter placed two targets 3 m apart and 3 m from the starting position (Figure 1B).

The experimenter started chewing mint gum and wore an FFP2-mask to keep from being influenced by the smells. Then, donning gloves, she opened and placed one tube in each plastic box at the back of the respective target, before disinfecting her hands. Each of the ten trials had two phases (Supplementary Video S1):

1. Guided phase: The experimenter walked the leashed dog to one target, allowing the dog to sniff at least 3 s in target proximity (50 cm) before repeating the same at the second target. The order was counterbalanced across trials.
2. Test phase: The unleashed dog was positioned parallel to the experimenter, facing the targets. The experimenter gave the command while looking straight and pointing exactly between the targets. The dog was verbally rewarded by the experimenter upon executing the command at whatever target it chose, called back, given a food reward (kibble or sausage), leashed, and returned to the owner.

The command was repeated up to five times if the dog moved toward the target but did not approach it within 20 cm before looking at the experimenter. If the dog did not move forward at all for three consecutive commands in a row, the trial was likewise terminated.

Targets were repositioned between trials, either exchanging their side or just shortly lifting them in place (sequence counterbalanced within and across dogs). Terminated trials were not repeated. If the dog did not approach the target three trials in a row, the session was terminated.

### 2.7 Behavioral variables

The test session was filmed, and behaviors were coded using BORIS software [v.8.25.4, (38)]. Interactions with the targets were analyzed in both phases, whereas choice behaviors, as well as experimenter- and environment-directed behaviors, were only coded in the Test phase (Table 1). Displacement signals (e.g., yawning, nose licking) could not be reliably coded due to camera positioning. All videos were coded blind to condition, group, and smell identity, and 21% (13 videos) were re-coded by a second coder who was blind to hypotheses and group allocation, achieving an interrater agreement of 0.95 (ICC 0.82–1.00; see Supplementary Table A).

**Table 1:** Ethogram.

Behavior	Definition
Guided phase	
Looking at target (D)	Time spent with the head directed toward target
Sniffing target (D)	Time spent with the nose within 10 cm of the target while nose directed toward target
Proximity to target (D)	Time spent with the nose within 50 cm circle around the target
Test phase	
Proximity to target (D)	Time with the nose within 50 cm circle around the target
Engaging with target (D)	Time spent with the head or paw within 10 cm of the target, gazing at it, sniffing, or touching it
Looking at target (D)	Time spent with the head directed toward target
Latency to accomplish command (D)	Time from first command to accomplishing it (touch for touch dogs, within 10 cm for close proximity dogs, half-way around target within 50 cm for running-around dogs)
Command accomplished (F/B)	Command (definition above) accomplished at target
Not accomplished (F/B)	Dog did not execute the command at all in that trial
Redirection (F)	Animal gets within 1 m of target with head oriented to the target, then executes the command at the other target
Number of commands (F)	Every time human gives a verbal and hand signal command
Tail high (D)	Time spent with the majority of the tail held at mid-point or above body line
Tail low (D)	Time spent with the majority of the tail held below body line
Looking at experimenter (D)	Time spent with the head directed toward experimenter
Proximity to experimenter (D)	Time with at least one paw within 1 m of the experimenter
Sniffing environment (D)	Nose within 10 cm of floor, directed at floor
Trial duration	Time from first command until dog returns to experimenter (first paw within 50 cm + remains there)

Behaviors were coded as durations (D), frequencies (F), or binary (B) in the two phases (Guided/Test) of each trial. Target-directed behaviors were coded as directed at the left or right target and later cross-referenced to the smell currently located on the respective side for analysis.

### 2.8 Statistical analysis

Analyses were conducted in R [RStudio v2023*0.06.0; (39)]. Duration variables were analyzed as proportions of the trial duration or time in target proximity. Low-frequency behaviors were analyzed as binary occurrences per trial. Behaviors occurring in less than 10% of trials were excluded. Target-directed behaviors were first analyzed as GLMMs with Smell and Group as main interaction factors, Sex, Age, and Trial (both z-transformed) as control factors, and AnimalID as a random effect. For the Smell factor, the first neutral movie sample represented Smell 1, while the second movie sample (neutral for control, fear for experimental group) was Smell 2. To further investigate if and why some dogs may react differently to the fear smell than others, we additionally analyzed the target-directed behaviors in the experimental group as an interaction of Smell and Sex as well as Smell and Age, with Trial as a control factor and AnimalID as a random factor. Non-target-directed behaviors were analyzed with the experimental group in interaction with Age and Sex, respectively, as the factor of interest. Random slopes were manually dummy-coded and centered. Relative durations were analyzed using a beta distribution, frequencies were modeled with a Poisson distribution, with the binomial model as the binary choice, with total trial number as offset and latency as a Gaussian family (log-transformed). Models were examined for overdispersion, distribution of residuals, Best Linear Unbiased Prediction, multicollinearity, and model stability. To keep the type I error rate at 5%, only significant variables in models that passed the full-null model comparisons using a likelihood ratio test (40) were examined in Tukey-adjusted pairwise comparisons (emmeans package (41)). Confidence intervals were obtained through Parametric bootstrapping (glmmTMB package (42)). Detailed model outputs are reported in the Supplementary material. A Fisher’s test determined whether test session termination or side biases occurred significantly more frequently in the experimental vs. control group. To analyze the strength of individual differences, mean target-directed behavior values were calculated per smell and individual, and the absolute difference between smells was computed for each individual. Due to non-parametric distribution, Mann–Whitney U tests were used to compare the strength of absolute differences between groups.

All utilized analyses were suitable for imbalanced sample sizes (43–45).

## 3 Results

**Figure 2:**
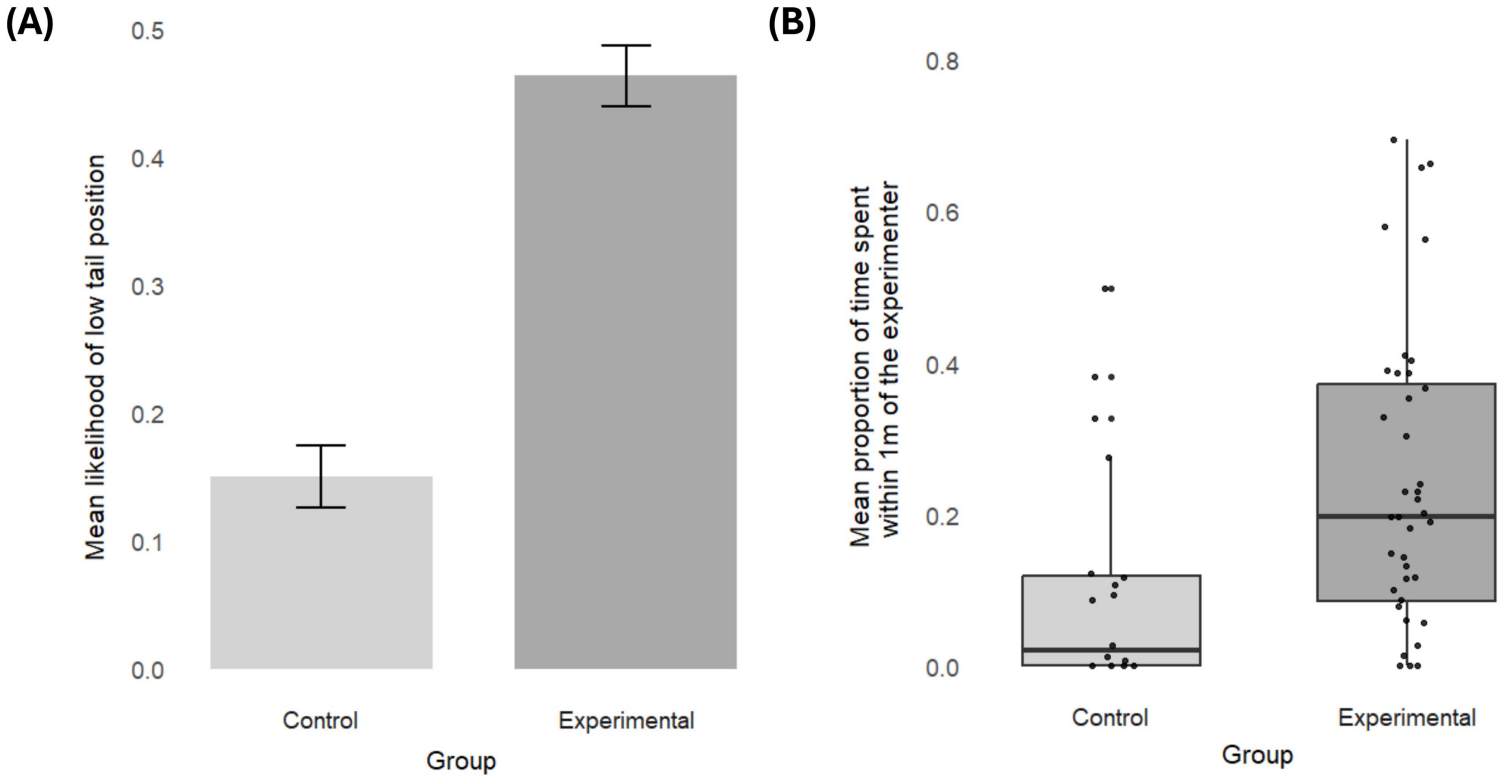
Group differences. **(A)** Mean likelihood of holding the tail low in each group. The bars represent the standard error. **(B)** Mean proportion of time spent within proximity (1 meter) of the experimenter in each group. Each box represents the interquartile range (25th–75th percentile) of the behavior, with the median marked by the thick line. Whiskers extend to the smallest and largest values within 1.5 times the interquartile range. Individuals are represented as black dots.

Dogs in the experimental group spent more time within 1 m of the experimenter (Control vs. experimental: est. = − 0.17, SE = 0.07, z.ratio = −2.51, *p* = 0.01) and were more likely to hold their tail in a position below the midpoint (control vs. experimental: est. = −1.79, SE = 0.87, z.ratio = −2.06, *p* = 0.04) (Figure 2). There was no difference between groups in how long the dogs held their tail up (χ^2^ = 4.38, df = 5, *p* = 0.50), looked at the experimenter (χ^2^ = 1.5, df = 5, *p* = 0.90), or sniffed the ground (χ^2^ = 8.78, df = 5, *p* = 0.12). Group did not affect how often they did not accomplish a trial (χ^2^ = 2.00, df = 5, *p* = 0.85), but ten dogs (out of 41) stopped to participate entirely in the experimental group compared to one dog (out of 20) in the control group, which was a marginal effect (Fisher’s Exact Test, *p* = 0.08). The frequency of redirection was too infrequent to be analyzed (10/515 trials). No effect of age or sex emerged for the group differences, neither as an interaction with group nor as an additive effect (see Supplementary material).

There was no choice preference for a certain smell in either group, neither in the first trial (χ^2^ = 4.88, df = 3, *p* = 0.18) nor across all trials (χ^2^ = 4.29, df = 6, *p* = 0.64) (see Table 2 for choice overview). On average, dogs in both groups chose one side 47% more often than the other. The probability of side bias did not differ significantly between groups (Fisher’s exact test, *p* = 0.53), with 45% of dogs in the Control group and 36% of dogs in the experimental group choosing the same side in at least 80% of trials (i.e., above chance at *p* = 0.055, assuming 10 trials). For details per dog, see Supplementary Table B.

**Table 2:** Choice behavior.

Group	Smell (mean across all trials ± SD)	Smell (total in first trial)	Side (mean across all trials ± SD)	Side (total in first trial)
Experimental	Smell 1 (neutral) 44% ± 16%	Smell 1 (neutral) *n* = 18	Left 50% ± 30%	Left *n* = 26
	Smell 2 (fear) 46% ± 17%	Smell 2 (fear) *n* = 23	Right 40% ± 29%	Right *n* = 15
	No choice 10% ± 18%		Dogs showing bias (>80%) 45%	
	Preference Smell 2 > Smell 1 2% ± 27%		Preference for one side over the other 47% ± 32%	
Control	Smell 1 (neutral) 44% ± 13%	Smell 1 (neutral) *n* = 7	Left 35% ± 27%	Left *n* = 7
	Smell 2 (neutral) 50% ± 16%	Smell 2 (neutral) *n* = 13	Right 59% ± 29%	Right *n* = 13
	No choice 6% ± 11%		Dogs showing bias (≥80%) 36%	
	Preference Smell 2 > Smell 1 6% ± 26%		Preference for one side over the other 47% ± 36%	

Summary of dogs’ choice behavior in the experimental and control group for a certain side or smell in the first trial and across all trials.

Regarding target-directed behaviors, there was no difference between smells or groups for sniffing (χ^2^ = 1.63, df = 3, *p* = 0.65), engaging with (χ^2^ = 0.31, df = 3, *p* = 0.96), looking at (χ^2^ = 1.21, df = 3, *p* = 0.75), or staying close to either target (χ^2^ = 1.86, df = 3, *p* = 0.60). Similarly, the number of commands needed to accomplish the task did not differ (χ^2^ = 2.20, df = 3, *p* = 0.53). However, dogs in the experimental group tended to be slower to approach the target compared to the control group, though there was no interaction with Smell on a group level (Group effect: χ^2^ = 83.93, df = 3, *p* < 0.01, *post-hoc* control vs. experimental group: est. = − 0.19, SE = 0.11, z.ratio = −1.72, *p* = 0.08). Neither sex nor age showed an interaction effect with smell in the experimental group (for outcome details see Supplementary material), but an additive effect emerged across groups, wherein age was negatively correlated with time spent sniffing the target (est. = − 0.21, SE = 0.08, z.ratio = −2.47, *p* = 0.013).

**Figure 3:**
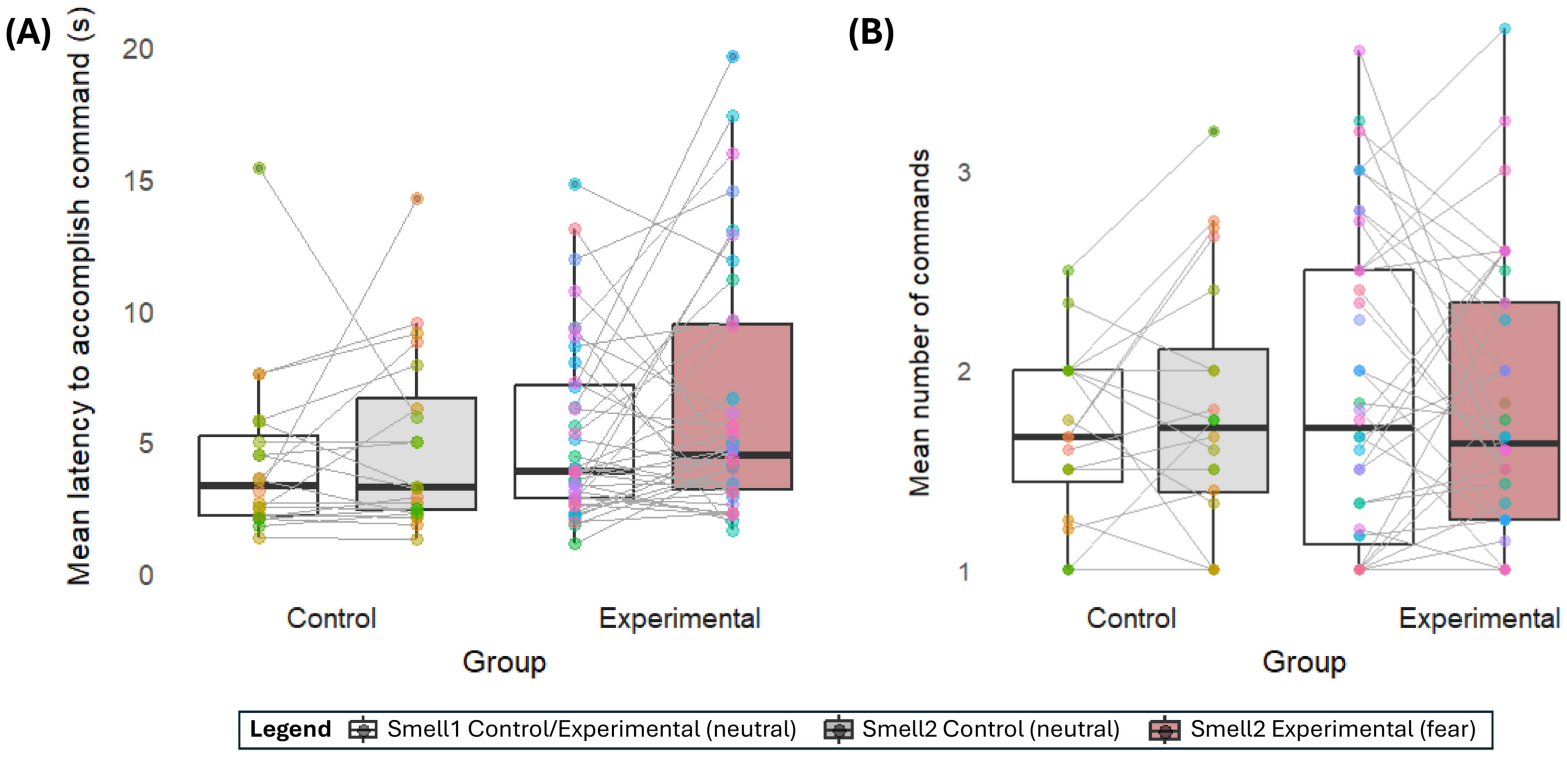
Individual differences. The mean **(A)** latency and **(B)** number of commands a dog needed in each group to approach Smell 1 [neutral (white)] or Smell 2 [Control group: neutral (grey), experimental group: fear (brown)]. Each individual animal is represented by a color consistent between both smells, connected by a grey line. The steeper the line, the stronger the individual’s preference to show the behavior at one smell target over the other. E.g., a steep upward line in the experimental group in **(A)** suggests the individual approached the fear smell much slower than the neutral human smell.

Similarly, dogs in the experimental group did not show bigger individual differences in how long they engaged with the two smells (W = 403, *p* = 0.92), sniffed them (W = 460, *p* = 0.45), looked at them (W = 400, *p* = 0.89), stayed in proximity to them (W = 347, *p* = 0.34), or which one they chose (W = 449, *p* = 0.54) compared to the control group. However, compared to the control group, dogs in the experimental group showed stronger individual differences in how quickly they approached one smell compared to the other (W = 262, *p* = 0.02) and a trend in the mean number of commands they needed to approach one smell compared to the other (W = 302, *p* = 0.09) (Figure 3).

## 4 Discussion

Studies across the last decade suggest that dogs react to human emotional chemosignals (11, 22, 23), but confirmation of the direct effect of the chemosignals in the absence of the potential reaction of simultaneously exposed humans to the scent was lacking. Although we did not find an overall preference for the fear or neutral scent, the presence of the human fear smell affected dogs’ behavior similarly to previous studies, even while the human present was shielded from the chemosignals. Moreover, it seems that the focus on between-group comparisons in previous studies might have masked subtle differences in individual reactions to human fear chemosignals.

In detail, dogs exposed to the fear smell spent more time near the experimenter, took longer to approach the target, were more likely to hold their tail low, and tended to disengage from the session (marginally) more frequently than dogs in the control group. D’Aniello and colleagues interpreted the proximity seeking, in their case, to the owner, as a safe-haven effect (21, 22), where dogs seek human attachment figures in threatening situations (46, 47). While our experimenter was not the owner, she was positioned closer to the owner than the targets and had previously interacted with the dog in rewarding training sessions, rendering her more familiar than the strangers in previous tests. This might explain dogs’ pronounced proximity-seeking in the experimental group compared to the control group, suggesting dogs’ discomfort or uncertainty in the presence of the fear smell. Dogs’ decreased willingness to approach the targets and the higher likelihood of disengagement align with that interpretation, mirroring dogs’ disinclination to approach strangers (11, 21) or ambiguous stimuli when exposed to human fear smell (23). Consistent with previous results, we also found a higher likelihood of a lowered tail posture (48, 49). This behavior, taken alone, may either be an expression of a relaxed or disinterested state or indicate more negative affect. However, we suggest that, integrated with our other results, the discomfort explanation is more likely. The consistency of these outcomes, despite the human being shielded from the smell, provides further and stronger evidence that dogs distinguish and react to the presence of human fear smell in the environment with behaviors indicating low-level discomfort or hesitation.

While we cannot entirely rule out that the human was not influenced by the smell, we believe our methodological precautions excluded this possibility. The test was conducted outdoors, reducing scent detectability (50). The experimenter wore an FFP-mask, which significantly decreases olfactory sensitivity and increases detection thresholds (30, 31). Furthermore, she chewed mint gum, increasing volatile concentration and inducing positive affect (51). Given that humans have significantly higher detection thresholds than dogs, especially for animalistic smells (52), we are confident that human influence was unlikely.

Having said that, our results suggest a more complex situation than direct interspecific olfactory-mediated emotional contagion. Moving beyond spontaneous behavior and between-group comparisons (11, 21, 22), our choice paradigm required dogs to take an action, eliciting greater variability and the possibility to robustly explore variation within and between subjects and smells. Therein, dogs in the experimental group showed stronger interindividual variation in their latency and the number of needed commands to approach the smells than the control group. The slopes in Figure 3 underline that, while some dogs in the experimental group hesitated to approach the fear sample, others approached it faster than the neutral sample. This variability contradicts the idea of a uniform, inherent avoidance of human fear scent in dogs (22). However, these results fit previous findings that dogs sniff human fear with the left nostril (rather than the right nostril when sniffing dog fear) (17), which is connected to threat-validity analysis (left-hemisphere) rather than a direct threat response (right hemisphere). This finding could suggest that dogs’ life experience may impact dogs’ reaction to human fear smell, for example, by associatively learning that the smell of fear means something negative (e.g., the owner yanking the leash) or positive (e.g., the owner petting the dog for personal stress relief), or through training that outside stimuli beyond the task should be ignored completely. Interestingly, similarly to previous studies, neither age nor sex predicted how dogs reacted to the fear compared to the control smell in the experimental group (21). On the other hand, Siniscalchi, d’Ingeo (17) found that dogs’ predatory behavior was correlated with how much they used the left nostril to smell human fear, leading the authors to hypothesize that dogs’ prey drive may modulate how much they choose to approach interspecific fear smell. Unfortunately, life experiences, training backgrounds, and breeds were too varied in our sample to be analyzed as possible explanators. Future studies with more uniform, dedicated recruitment will be needed to elucidate the underlying drivers. Furthermore, the area in which dogs are exposed to the smell may play a role in their reaction. We had balanced our experimental and control groups across the test locations (Clever Dog Lab and dog school), both chosen as locations where the participating dogs had a comparatively variable experience in how often they had been there before and what kind of tasks they engaged in. For the sake of model complexity and small sample tested outside the lab (*n* = 7/61 at dog school, *n* = 1 at home), location was not added to the analysis, and no descriptive differences emerge from the data (see Supplementary Table B). However, future studies may want to explore whether familiar and unfamiliar location influences dogs’ reactions toward human fear chemosignals differently.

Since we were unable to code the displacement behaviors, we cannot make any claims about discomfort beyond what matched the behaviors from previous studies. Hence, it needs to be further explored whether the overall slower approach in the experimental group compared to the control group was a remnant of innate fear avoidance, whether it was discomfort at all, or whether it may reflect initial uncertainty before determining whether the approach was safe or allowed. Given the correlation to affect, monitoring dogs’ lateralized behavior in, for example, their tail wagging direction or paw or nostril use, may help further clarify, beyond simple approach metrics, why each dog chose a certain behavior toward the human fear smell (17, 53–55).

While the finding of significant individual preferences for one smell over the other in the latency to approach but not the choice itself might seem puzzling, this pattern aligns with prior findings in dogs’ free choice behavior. Beyond the ubiquitous problem of side biases in choice tests (56, 57), recency effects significantly influence decision-making in dogs (58). While trained dogs excel at scent detection, untrained dogs tend to rely on win-stay/loose-shift strategies when choosing between familiar and unfamiliar stimuli (37, 58). Since our dogs were rewarded for any choice, this factor likely influenced the behavior in our sample.

Taken together, dogs exposed to the human fear smell displayed more negative affect and reluctance to leave the human and approach either target, strengthening previous findings that dogs react to human fear smell even when the human is unaffected. Despite this, some dogs avoided the human fear smell, while others approached it faster than the neutral smell, suggesting subtle individual differences in how dogs react to human fear chemosignals. Age and sex did not explain this pattern. Our results emphasize that exploring variability in dogs’ reactions rather than assuming uniformity is crucial when researching companion dog behavior. Studies to validate this outcome and explore possible drivers are clearly needed. Better understanding why and whether a dog approaches or avoids human fear may aid our interactions with dogs all from safety (e.g., attacks on fearful people), welfare (e.g., decreasing overall dog stress), and practical (e.g., selecting therapy dogs) perspectives.

## Data Availability

The original contributions presented in the study are included in the article/Supplementary material, further inquiries can be directed to the corresponding authors.
