# Supplementary material for: Not just avoidance: dogs show subtle individual differences in reacting to human fear chemosignals
Source: Front Vet Sci. 2025 Sep 15;12:1679991. doi: 10.3389/fvets.2025.1679991 (PMC12477697; doi:10.3389/fvets.2025.1679991)
Supplement: Supplementary file 4 [file Data_Sheet_1.PDF]

```
#-#-#-#-#-#-#-#-#-#-#-#-#-#-#-#-#-#-#-#-#-#-#-#-#-#-#-#-#-#-#-#-#-#-#-#-#--#-#
#                                                                              #
#    R-Analysis script for:                                                    #
#    Not just avoidance: Dogs show subtle individual differences               #
#            in reacting to human fear chemosignals                            #
#                                                                              #
#    Svenja Capitain, Friederike Range, Sarah Marshall-Pescini                 #
#            Frontiers in Veterinary Science, 2025                             #
#                                                                              #
#-#-#-#-#-#-#-#-#-#-#-#-#-#-#-#-#-#-#-#-#-#-#-#-#-#-#-#-#-#-#-#-#-#-#-#-#--#-#


#-#-#-#-#-#-#-#-#-#-#-#-#-#-#-#-#-#-#-#-#-#-#-#
#    Load and install packages       #####
#-#-#-#-#-#-#-#-#-#-#-#-#-#-#-#-#-#-#-#-#-#-#-#

library(dplyr)
library(tidyr)
library(ggplot2)
library(lme4)
library(glmmTMB)
library(DHARMa)
library(car)
library(coxme)
library(survival)
library(emmeans)
library(boot)
library(caret)
library(lmtest)
library(ggbeeswarm)

source(".../diagnostic_fcns.r")
source(".../boot_glmm.r")
source(".../glmmTMB_stability.r")
source(".../glmm_stability.r")



#-#-###-#-#-#-#-#-#-#-#-#-#-#-#-#-#-#-##-
# 1. Load Data                ----
#-#-###-#-#-#-#-#-#-#-#-#-#-#-#-#-#-#-##-

# Data file (as csv): "Capitain2025_FVets_ScentPreference_InputData_GroupComparison.csv"
combined_table <- data.frame(read.csv(file.choose(), sep = ",", dec = ".", header = T,
stringsAsFactors=T))

str(combined_table)

combined_table$Age <- as.numeric(combined_table$Age)

#-#-#-#-#-#-#-#-#-#-#-#-#-#-#-#-#-#-#-#-#-#-#-#-#
# 2. Models  -  Group*Smell interaction   ----
#-#-#-#-#-#-#-#-#-#-#-#-#-#-#-#-#-#-#-#-#-#-#-#-#
```

```
#________________________#
## * Choice        ----
#________________________#

# Reduce database to command accomplished on a certain side
choice <- combined_table %>%
  mutate(Command.accomplished = ifelse(Side.Smell2 == "", NA, Command.accomplished ))
choice <- choice %>%  filter(Command.accomplished != "NA")

# check for random slopes - - - - - - - - - - -
xx.fe.re=fe.re.tab(fe.model="Command.accomplished ~ Smell * Group + Age + Sex + Trial +
TotalChoices",
             re="(1|AnimalID)", data=choice)
summary(xx.fe.re)

#factors are already dummycoded in fe.re. function
choice=xx.fe.re$data #place in new object

# z-transform numeric predictors
choice$Trial<-as.numeric(choice$Trial)
choice$Age.z<-as.vector(scale(choice$Age))
choice$Trial.z<-as.vector(scale(choice$Trial))
choice$TotalChoices_log <- log(choice$TotalChoices)


# model- - - - - - - - - - -
full.Choice=glmer(Command.accomplished ~ Smell*Group + Trial.z + Age.z + Sex +
             offset(TotalChoices_log) +
              (1+Smell.Smell2+Trial.z|AnimalID),
              family=binomial,
              data=choice,
              glmerControl(optimizer="bobyqa", optCtrl=list(maxfun=2e5)))
summary(full.Choice)

#check assumptions - - - - - - - - - - -
#Collinearity
full.Choice.m.coll=lme4::lmer(Command.accomplished ~ Smell+Group + Age.z + Sex + Trial.z +
                   (1+Smell.Smell2+Trial.z||AnimalID),
                  data=choice)
round(vif(full.Choice.m.coll), 3) # fine if below 3
#Model stability
bin.stab <- glmm.model.stab(model.res = full.Choice)
bin.stab$summary[, -1]
m.stab.plot(bin.stab$summary[, -1]) # good


#Null-Full model comparison  - - - - - - - - - - - - - - -
null.Choice=glmer(Command.accomplished ~ Age.z + Sex + Trial.z +
             offset(TotalChoices_log) +
             (1+Smell.Smell2+Trial.z||AnimalID),
            family=binomial,
            data=choice,
```

```
        glmerControl(optimizer="bobyqa", optCtrl=list(maxfun=2e5)))

summary(null.Choice)

anova_full.Choice <- anova(null.Choice,full.Choice)
print(anova_full.Choice) # ns

#plot trial effect
ggplot(choice, aes(x = Trial.z, y = Command.accomplished, color = Group)) +
  geom_jitter(width = 0.2, height = 0.05, alpha = 0.4) +  # Scatter points
  geom_smooth(method = "glm", method.args = list(family = "binomial"), se = TRUE) +  # Logistic
regression
  scale_color_manual(values = c("Control" = "blue", "Experimental" = "red")) +  # Custom colors
  labs(x = "Trial (z-scored)",
     y = "Probability of Choosing Smell2",
     title = "Effect of Trial on Choosing Smell2 by Group") +
  theme_minimal()

# Confidence intervals
boot.bin.Choice=boot.lmer(m=full.Choice, discard.warnings=F, nboots=100, para=F,
              resol=1000, level=0.95, use=c("task", "age"))
boot.bin.Choice$ci.estimates
m.stab.plot(boot.bin.Choice$ci.estimates) #not pretty -> small sample size


#________________________#
## * First Choice       ----
#________________________#

# Reduce database to command accomplished on a certain side
FiChoice <- combined_table %>%
  filter(TrialNo == "Test1") %>%
  mutate(Command.accomplished = ifelse(Side.Smell2 == "", NA, Command.accomplished ))

FiChoice <- FiChoice %>%  filter(Command.accomplished != "NA")

# check for random slopes - - - - - - - - - - -
xx.fe.re=fe.re.tab(fe.model="Command.accomplished ~ Smell * Group + Age + Sex + Trial",
          re="(1|AnimalID)", data=FiChoice)
summary(xx.fe.re)

#factors are already dummycoded in fe.re. function
FiChoice=xx.fe.re$data #place in new object

# z-transform numeric predictors
FiChoice$Age.z<-as.vector(scale(FiChoice$Age))

# model- - - - - - - - - - -
full.FiChoice=glmer(Command.accomplished ~ Smell*Group + Age.z + Sex +
          (1+Smell.Smell2||AnimalID),
        family=binomial,
        data=FiChoice,
        glmerControl(optimizer="bobyqa", optCtrl=list(maxfun=2e5)))
```

```
summary(full.FiChoice)

#check assumptions - - - - - - - - - - -
#Collinearity
full.FiChoice.m.coll=lme4::lmer(Command.accomplished ~ Smell+Group + Age.z + Sex +
                     (1+Smell.Smell2||AnimalID),
                    data=FiChoice)
round(vif(full.FiChoice.m.coll), 3) # fine if below 3
#Model stability
bin.stab.fc <- glmm.model.stab(model.res = full.FiChoice)
bin.stab.fc$summary[, -1]
m.stab.plot(bin.stab.fc$summary[, -1]) # good


#Null-Full model comparison  - - - - - - - - - - - - - - - -
null.FiChoice=glmer(Command.accomplished ~ Age.z + Sex +
              (1+Smell.Smell2||AnimalID),
             family=binomial,
             data=FiChoice,
             glmerControl(optimizer="bobyqa", optCtrl=list(maxfun=2e5)))

summary(null.FiChoice)

anova_full.FiChoice <- anova(null.FiChoice,full.FiChoice)
print(anova_full.FiChoice) # ns

# Confidence intervals
boot.bin.FiChoice=boot.lmer(m=full.FiChoice, discard.warnings=F, nboots=100, para=F,
                 resol=1000, level=0.95, use=c("task", "age"))
boot.bin.FiChoice$ci.estimates
m.stab.plot(boot.bin.FiChoice$ci.estimates) #not pretty -> small sample size



#__________________________________________#
## * Side Preference Choice         ----
#__________________________________________#

# Reduce database to command accomplished on a certain side
side <- combined_table %>%
  mutate(Command.accomplished = ifelse(Side.Behaviour == "", NA, Command.accomplished ))
side <- side %>%  filter(Command.accomplished != "NA")

# check for random slopes - - - - - - - - - - -
xx.fe.re=fe.re.tab(fe.model="Command.accomplished ~ Side.Behaviour * Group + Age + Sex + Trial",
             re="(1|AnimalID)", data=side)
summary(xx.fe.re)

#factors are already dummycoded in fe.re. function
side=xx.fe.re$data #place in new object

# z-transform numeric predictors
side$Trial<-as.numeric(side$Trial)
```

```
side$Age.z<-as.vector(scale(side$Age))
side$Trial.z<-as.vector(scale(side$Trial))


# model- - - - - - - - - - - -
full.side=glmer(Command.accomplished ~ Side.Behaviour*Group + Age.z + Sex + Trial.z +
          (1+Trial.z||AnimalID),
         family=binomial,
         data=side,
         glmerControl(optimizer="bobyqa", optCtrl=list(maxfun=2e5)))
summary(full.side)

#check assumptions - - - - - - - - - - -
#Collinearity (take out interactions)
full.side.m.coll=lme4::lmer(Command.accomplished ~ Side.Behaviour+Group + Age.z + Sex + Trial.z +
                (1+Trial.z||AnimalID),
               data=side)
round(vif(full.side.m.coll), 3) # fine if below 3
#Model stability
bin.stab <- glmm.model.stab(model.res = full.side)
bin.stab$summary[, -1]
m.stab.plot(bin.stab$summary[, -1]) #good


#Null-Full model comparison  - - - - - - - - - - - - - - - -
null.side=glmer(Side.Behaviour ~ Age.z + Sex + Trial.z +
          (1+Trial.z||AnimalID),
         family=binomial,
         data=side,
         glmerControl(optimizer="bobyqa", optCtrl=list(maxfun=2e5)))

summary(null.side) #converged

anova_full.side <- anova(null.side,full.side)
print(anova_full.side) # highly significant

#Test for each predictor  - - - - - - - - - - - - - - - -
drop1(full.side, test="Chisq")
# Interaction significance

emmeans(full.side, pairwise ~ Side.Behaviour*Group)


#________________________#
## * Proximity        ----
#________________________#

# Update the proximity column based on the condition in Side.Smell2
proximity <- combined_table %>%
  mutate(Proximity.to.target..G.T. = ifelse(Side.Smell2 == "", NA, Proximity.to.target..G.T. ))
proximity <- proximity %>%  filter(Proximity.to.target..G.T. != "0")
proximity <- proximity %>%  filter(Proximity.to.target..G.T. != "NA")
proximity <- proximity %>%  filter(TrialType == "Test")
```

```
# check for random slopes - - - - - - - - - - -
xx.fe.re=fe.re.tab(fe.model="Proximity.to.target..G.T. ~ Smell * Group + Age + Sex + Trial",
            re="(1|AnimalID)", data=proximity)
summary(xx.fe.re)

#factors are already dummycoded in fe.re. function
proximity=xx.fe.re$data #place in new object

# z-transform numeric predictors
proximity$Trial<-as.numeric(proximity$Trial)
proximity$Age.z<-as.vector(scale(proximity$Age))
proximity$Trial.z<-as.vector(scale(proximity$Trial))

hist(log(proximity$Proximity.to.target..G.T.+1))
proximity$Proximity.to.target_log <- log(proximity$Proximity.to.target..G.T.+1)

# Model
full.proximity <- lmer(Proximity.to.target_log ~ Smell * Group + Age.z + Sex + Trial.z +
                      (1+Smell.Smell2+Trial.z|AnimalID),
                      data = proximity)
summary(full.proximity)
summary(full.proximity)$varcor

#check assumptions - - - - - - - - - - -
sim_res <- simulateResiduals(fittedModel = full.proximity)
plot(sim_res)
ranef.diagn.plot(full.proximity) #BLUPS, all normally distributed, range small
#Collinearity (take out interactions)
full.ProxP.m.coll=lme4::lmer(Proximity.to.target_log  ~ Smell + Group + Age.z + Sex + Trial.z +
                      (1+Smell.Smell2+Trial.z|AnimalID),
                     data=proximity)
round(vif(full.ProxP.m.coll), 3) # fine if below 2
#Model stability
full.stab.proximity=glmm.model.stab(model.res=full.proximity, contr=NULL,para=F, data=NULL)
m.stab.plot(full.stab.proximity$summary[, -1])  # for fixed effects


#Null-Full model comparison  - - - - - - - - - - - - - - - -
null.proximity=lmer(Proximity.to.target_log ~ Age.z + Sex + Trial.z +
             (1+Smell.Smell2+Trial.z|AnimalID),
           data=proximity)

summary(null.proximity) #not converged

anova_full.proximity <- anova(null.proximity,full.proximity)
print(anova_full.proximity) # ns
summary(full.proximity) # rest also ns

#confidence intervals
boot.full.proximity=boot.lmer(m=full.proximity, discard.warnings=F,
                   nboots=1000, para=T, n.cores=6, resol=1000, level=0.95)
round(boot.full.proximity$ci.estimates, 3) #extract them
```

```
m.stab.plot(boot.full.proximity$ci.estimates)

#________________________#
## *  Sniffing target       ----
#________________________#

# Update the sniffing column based on the condition in Side.Smell2
sniffingTP <- combined_table %>%
  mutate(Sniffing.target.PropP = ifelse(Side.Smell2 == "", NA, Sniffing.target.PropP ))
sniffingTP <- sniffingTP %>%  filter(Sniffing.target.PropP != "NA")

# check for random slopes - - - - - - - - - - -
xx.fe.re=fe.re.tab(fe.model="Sniffing.target.PropP ~ Smell * Group + Age + Sex + Trial",
               re="(1|AnimalID)", data=sniffingTP)
summary(xx.fe.re)

#factors are already dummycoded in fe.re. function
sniffingTP=xx.fe.re$data #place in new object

# z-transform numeric predictors
sniffingTP$Trial<-as.numeric(sniffingTP$Trial)
sniffingTP$Age.z<-as.vector(scale(sniffingTP$Age))
sniffingTP$Trial.z<-as.vector(scale(sniffingTP$Trial))

#transform because beta cannot handle 0 and 1!!!
sniffingTP$Sniffing.target.PropP=beta.tr(sniffingTP$Sniffing.target.PropP)
sniffingTP$Sniffing.target.PropP  <- ifelse(sniffingTP$Sniffing.target.PropP  > 1, 0.9999,
sniffingTP$Sniffing.target.PropP)

hist((sniffingTP$Sniffing.target.PropP)) #it's a proportion, they can look weird. Just has to be bound
beweeen 0 and 1

# Model
full.sniffingTP <- glmmTMB(Sniffing.target.PropP ~ Smell * Group + Age.z + Sex + Trial.z +
                      (1+Smell.Smell2+Trial.z||AnimalID),
                     family = beta_family,
                     data = sniffingTP)

summary(full.sniffingTP)
summary(full.sniffingTP)$varcor

#check assumptions - - - - - - - - - - -
ranef.diagn.plot(full.sniffingTP) #BLUPS, all normally distributed, range small
#Collinearity (take out interactions)
full.SniffTP.m.coll=lme4::lmer(Sniffing.target.PropP ~ Smell + Group + Age.z + Sex + Trial.z +
                         (1+Smell.Smell2+Trial.z||AnimalID),
                       data=sniffingTP)
round(vif(full.SniffTP.m.coll), 3) # fine if below 2
#Model stability
stab.full.SniffTP=glmmTMB.stab(model.res=full.sniffingTP, para=T,
                      data=sniffingTP)
table(stab.full.SniffTP$detailed$converged)
m.stab.plot(stab.full.SniffTP$summary[, -1]) #good
```

```
#Null-Full model comparison - - - - - - - - - - - - - - - -
null.SniffTP=glmmTMB(Sniffing.target.PropP ~ Age.z + Sex + Trial.z +
                    (1+Smell.Smell2+Trial.z||AnimalID),
                   family = beta_family,
                   data = sniffingTP)

summary(null.SniffTP) #converged

anova_full.SniffTP <- anova(null.SniffTP,full.sniffingTP)
print(anova_full.SniffTP) # not significant

#inference
drop1(full.sniffingTP, test="Chisq")
summary(full.sniffingTP)

#confidence intervals
boot.full.sniffingTP=boot.glmmtmb(m=full.sniffingTP, data=sniffingTP,
                      discard.non.conv=F, nboots=1000, para=T, resol=100,
                      level=0.95, n.cores="all-1") #all-1 leaves you capacity on teh laptop to do other
things

boot.full.sniffingTP$ci.estimates$fe
m.stab.plot(boot.full.sniffingTP$ci.estimates$fe, 3)

# Age effect
ggplot(combined_table,aes(Age, Sniffing.target.PropP)) +
  stat_summary(fun.data=mean_cl_normal) +
  geom_smooth(method='lm', formula= y~x)


#__________________________#
## * Engaging target       ----
#__________________________#

# Update the column based on the condition in Side.Smell2
EngagingTP <- combined_table %>%
  mutate(Engaging.with.target.PropP = ifelse(Side.Smell2 == "", NA, Engaging.with.target.PropP ))
EngagingTP <- EngagingTP %>%  filter(Engaging.with.target.PropP != "NA")
EngagingTP <- EngagingTP %>%  filter(Engaging.with.target.PropP != "0")

# check for random slopes - - - - - - - - - - -
xx.fe.re=fe.re.tab(fe.model="Engaging.with.target.PropP ~ Smell * Group + Age + Sex + Trial",
            re="(1|AnimalID)", data=EngagingTP)
summary(xx.fe.re)

#factors are already dummycoded in fe.re. function
EngagingTP=xx.fe.re$data #place in new object

# z-transform numeric predictors
EngagingTP$Trial<-as.numeric(EngagingTP$Trial)
EngagingTP$Age.z<-as.vector(scale(EngagingTP$Age))
EngagingTP$Trial.z<-as.vector(scale(EngagingTP$Trial))
```

```
#transform because beta cannot handle 0 and 1!!!
EngagingTP$Engaging.with.target.PropP=beta.tr(EngagingTP$Engaging.with.target.PropP)
EngagingTP$Engaging.with.target.PropP  <- ifelse(EngagingTP$Engaging.with.target.PropP  > 1,
0.9999, EngagingTP$Engaging.with.target.PropP)

hist((EngagingTP$Engaging.with.target.PropP))

# Model
full.EngagingTP <- glmmTMB(Engaging.with.target.PropP ~ Smell * Group + Age.z + Sex + Trial.z +
                          (1+Smell.Smell2+Trial.z||AnimalID),
                         family = beta_family,
                         data = EngagingTP)

summary(full.EngagingTP)
summary(full.EngagingTP)$varcor

#check assumptions - - - - - - - - - - -
ranef.diagn.plot(full.EngagingTP) #BLUPS, all normally distributed, range small
#Collinearity (take out interactions)
full.EngagTP.m.coll=lme4::lmer(Engaging.with.target.PropP ~ Smell + Group + Age.z + Sex + Trial.z +
                               (1+Smell.Smell2+Trial.z||AnimalID),
                             data=EngagingTP)
round(vif(full.EngagTP.m.coll), 3) # fine if below 2
#Model stability
stab.full.EngagTP=glmmTMB.stab(model.res=full.EngagingTP, para=T,
                             data=EngagingTP)
table(stab.full.EngagTP$detailed$converged)
m.stab.plot(stab.full.EngagTP$summary[, -1]) #good

#Null-Full model comparison  - - - - - - - - - - - - - - - -
null.EngagTP=glmmTMB(Engaging.with.target.PropP ~ Age.z + Sex + Trial.z +
                      (1+Smell.Smell2+Trial.z||AnimalID),
                    family = beta_family,
                    data = EngagingTP)

summary(null.EngagTP) #converged

anova_full.EngagTP <- anova(null.EngagTP,full.EngagingTP)
print(anova_full.EngagTP) # not significant

#confidence intervals
boot.full.EngagingTP=boot.glmmtmb(m=full.EngagingTP, data=EngagingTP,
                                discard.non.conv=F, nboots=1000, para=T, resol=100,
                                level=0.95, n.cores="all-1") #all-1 leaves you capacity on teh laptop to do other
things

boot.full.EngagingTP$ci.estimates$fe
m.stab.plot(boot.full.EngagingTP$ci.estimates$fe, 3)


#________________________#
## * Looking at target       ----
```

```
#_______________________#

# Update the column based on the condition in Side.Smell2
LookingTP <- combined_table %>%
  mutate(Looking.at.target.GT.Prop = ifelse(Side.Smell2 == "", NA, Looking.at.target.GT.Prop))

LookingTP <- LookingTP %>%  filter(Looking.at.target.GT.Prop != "NA")
LookingTP <- LookingTP %>%  filter(Looking.at.target.GT.Prop != "0")

# check for random slopes - - - - - - - - - - -
xx.fe.re=fe.re.tab(fe.model="Looking.at.target.GT.Prop ~ Smell * Group + Age + Sex + Trial",
            re="(1|AnimalID)", data=LookingTP)
summary(xx.fe.re)

#factors are already dummycoded in fe.re. function
LookingTP=xx.fe.re$data #place in new object

# z-transform numeric predictors
LookingTP$Trial<-as.numeric(LookingTP$Trial)
LookingTP$Age.z<-as.vector(scale(LookingTP$Age))
LookingTP$Trial.z<-as.vector(scale(LookingTP$Trial))

#transform because beta cannot handle 0 and 1!!!
LookingTP$Looking.at.target.GT.Prop=beta.tr(LookingTP$Looking.at.target.GT.Prop)
LookingTP$Looking.at.target.GT.Prop  <- ifelse(LookingTP$Looking.at.target.GT.Prop  > 1, 0.9999,
LookingTP$Looking.at.target.GT.Prop)

hist((LookingTP$Looking.at.target.GT.Prop))

# Model
full.LookingTP <- glmmTMB(Looking.at.target.GT.Prop ~ Smell * Group + Age.z + Sex + Trial.z +
                    (1+Smell.Smell2+Trial.z||AnimalID),
                   family = beta_family,
                   data = LookingTP)

summary(full.LookingTP)
summary(full.LookingTP)$varcor

#check assumptions - - - - - - - - - - -
ranef.diagn.plot(full.LookingTP) #BLUPS, all normally distributed, range small
#Collinearity (take out interactions)
full.LookTP.m.coll=lme4::lmer(Looking.at.target.GT.Prop ~ Smell + Group + Age.z + Sex + Trial.z +
                      (1+Smell.Smell2+Trial.z||AnimalID),
                     data=LookingTP)
round(vif(full.LookTP.m.coll), 3) # fine if below 2
#Model stability
stab.full.LookTP=glmmTMB.stab(model.res=full.LookingTP, para=T,data=LookingTP)
table(stab.full.LookTP$detailed$converged)
m.stab.plot(stab.full.LookTP$summary[, -1]) #good

#Null-Full model comparison  - - - - - - - - - - - - - - - -
null.LookTP=glmmTMB(Looking.at.target.GT.Prop ~ Age.z + Sex + Trial.z +
               (1+Smell.Smell2+Trial.z||AnimalID),
```

```
                  family = beta_family,
                  data = LookingTP)

summary(null.LookTP) #converged

anova_full.LookTP <- anova(null.LookTP,full.LookingTP)
print(anova_full.LookTP) # not significant

#confidence intervals
boot.full.LookingTP=boot.glmmtmb(m=full.LookingTP, data=LookingTP,
                         discard.non.conv=F, nboots=1000, para=T, resol=100,
                         level=0.95, n.cores="all-1") #all-1 leaves you capacity on teh laptop to do other
things

boot.full.LookingTP$ci.estimates$fe
m.stab.plot(boot.full.LookingTP$ci.estimates$fe, 3)


#________________________#
## * Commands needed        ----
#________________________#

# Create the 'command.smell' column, where command number value is placed in rows where
Command.accomplished == 1
combined_table <- combined_table %>%
  group_by(TrialNo, AnimalID) %>%
  mutate(
    Command.Smell = ifelse(
      Command.accomplished == 1,
      first(Number.of.commands[!is.na(Number.of.commands)]),
      NA )
  ) %>%
  ungroup()

# Update the column based on the condition in Side.Smell2
Commands <- combined_table %>%
  mutate(Command.Smell = ifelse(Side.Smell2 == "", NA, Command.Smell))
Commands <- Commands %>%  filter(Command.Smell != "NA")

# check for random slopes - - - - - - - - - - -
xx.fe.re=fe.re.tab(fe.model="Command.Smell ~ Smell * Group + Age + Sex + Trial",
               re="(1|AnimalID)", data=Commands)
summary(xx.fe.re)

#factors are already dummycoded in fe.re. function
Commands=xx.fe.re$data #place in new object

# z-transform numeric predictors
Commands$Trial<-as.numeric(Commands$Trial)
Commands$Age.z<-as.vector(scale(Commands$Age))
Commands$Trial.z<-as.vector(scale(Commands$Trial))

hist((Commands$Command.Smell))
```

```
# Model
full.Commands <- glmer(Command.Smell ~ Smell * Group + Age.z + Sex + Trial.z +
                        (1+Smell.Smell2+Trial.z||AnimalID), #doesn't converge with random slopes
                      family = poisson(),
                      data = Commands)
summary(full.Commands)
summary(full.Commands)$varcor

#check assumptions - - - - - - - - - - -
overdisp.test(full.Commands) # not overdispersed (not sign.)
ranef.diagn.plot(full.Commands) #BLUPS, all normally distributed, range small
#Collinearity (take out interactions)
full.Command.m.coll=lme4::lmer(Command.Smell ~ Smell + Group + Age.z + Sex + Trial.z +
                          (1+Smell.Smell2+Trial.z||AnimalID),
                        data=Commands)
round(vif(full.Command.m.coll), 3) # fine if below 2
#Model stability
bin.stab=glmm.model.stab(model.res=full.Commands)
bin.stab$summary[, -1]
m.stab.plot(bin.stab$summary[, -1]) #ok

#Null-Full model comparison  - - - - - - - - - - - - - - - -
null.Commands <- glmer(Command.Smell ~ Age.z + Sex + Trial.z +
                         (1+Smell.Smell2+Trial.z||AnimalID),
                       family = poisson(),
                       data = Commands)

summary(null.Commands) #converged

anova_full.Commands <- anova(null.Commands,full.Commands)
print(anova_full.Commands) # not significant


#confidence intervals
full.boot.Commands=boot.lmer(m=full.Commands,
                 nboots=100, para=F, n.cores=6, resol=100, level=0.95) #normally nboots=1000, para=T
but takes way longer

m.stab.plot(full.boot.Commands$ci.estimates)
full.boot.Commands$ci.estimates



#________________________#
## * Latency       ----
#________________________#

# Update the column based on the condition in Side.Smell2
Latency <- combined_table %>%
  mutate(LatencySmell = ifelse(Side.Smell2 == "", NA, LatencySmell))
Latency <- Latency %>%  filter(LatencySmell != "NA",
                         TrialType == "Test")
```

```
hist((Latency$LatencySmell))
hist(log(Latency$LatencySmell+1))
Latency$LatencySmell_log <- log(Latency$LatencySmell+1)

# check for random slopes - - - - - - - - - - -
xx.fe.re=fe.re.tab(fe.model="LatencySmell_log ~ Smell * Group + Age + Sex + Trial",
               re="(1|AnimalID)", data=Latency)
summary(xx.fe.re)

#factors are already dummycoded in fe.re. function
Latency=xx.fe.re$data #place in new object

# z-transform numeric predictors
Latency$Trial<-as.numeric(Latency$Trial)
Latency$Age.z<-as.vector(scale(Latency$Age))
Latency$Trial.z<-as.vector(scale(Latency$Trial))


# Model
full.lat <- lmer(LatencySmell_log ~ Group*Smell + Age.z + Sex + Trial.z +
               (1+Smell.Smell2+Trial.z||AnimalID),
                data = Latency)

summary(full.lat)

#check assumptions - - - - - - - - - - -
sim_res <- simulateResiduals(fittedModel = full.lat)
testDispersion(sim_res) # if SQRT, not overdispersed (not sign.)
#Collinearity (take out interactions)
full.latD.m.coll=lmer(LatencySmell_log ~ Group+Smell + Age.z + Sex + Trial.z +
              (1+Smell.Smell2+Trial.z|AnimalID),
              data = Latency)  # Body size and Species highly correlated
round(vif(full.latD.m.coll), 3) # fine if below 2
#Model stability
full.stab=glmm.model.stab(model.res=full.lat, contr=NULL,para=F, data=NULL)
m.stab.plot(full.stab$summary[-1])  # for fixed effects

#Null-Full model comparison  - - - - - - - - - - - - - - - -
null.lat=lmer(LatencySmell_log ~  Age.z + Sex + Trial.z +
               (1+Smell.Smell2+Trial.z||AnimalID),
             data = Latency)

summary(null.lat) #converged

anova_full.lat <- anova(null.lat,full.lat)
print(anova_full.lat) # significant

drop1(full.lat, test="Chisq")
# interaction ns
summary(full.lat)

#Reuced model
```

```
red.lat=lmer(LatencySmell_log ~ Group + Smell + Age.z + Sex + Trial.z +
             (1+Smell.Smell2+Trial.z||AnimalID),
            data = Latency)

summary(red.lat) #converged

drop1(red.lat, test="Chisq")

#check effects
emmeans(red.lat, pairwise ~ Group)


# Confidence interval
boot.red.Latency=boot.lmer(m=red.lat, discard.warnings=F,
                    nboots=1000, para=T, n.cores=6, resol=1000, level=0.95)
round(boot.red.Latency$ci.estimates, 3) #extract them
m.stab.plot(boot.red.Latency$ci.estimates)

boot.full.Latency=boot.lmer(m=full.lat, discard.warnings=F,
                     nboots=1000, para=T, n.cores=6, resol=1000, level=0.95)
m.stab.plot(boot.full.Latency$ci.estimates)
round(boot.full.Latency$ci.estimates, 3) #extract them



#-#-#-#-#-#-#-#-#-#-#-#-#-#-#-#-#-#-#-#-#-#-#-#
# 3. Models  -  Experimental Group: Smell*(Age+Sex) interaction   ----
#-#-#-#-#-#-#-#-#-#-#-#-#-#-#-#-#-#-#-#-#-#-#-#

combined_table.E <- combined_table %>%
  filter(Group == "Experimental")


#________________________#
## * Choice         ----
#________________________#

# Reduce database to command accomplished on a certain side
choice.E <- combined_table.E %>%
  mutate(Command.accomplished = ifelse(Side.Smell2 == "", NA, Command.accomplished ))
choice.E <- choice.E %>%  filter(Command.accomplished != "NA")

# check for random slopes - - - - - - - - - - -
xx.fe.re=fe.re.tab(fe.model="Command.accomplished ~ Smell * (Age + Sex) + Trial + TotalChoices",
               re="(1|AnimalID)", data=choice.E)
summary(xx.fe.re)

#factors are already dummycoded in fe.re. function
choice.E=xx.fe.re$data #place in new object

# z-transform numeric predictors
choice.E$Trial<-as.numeric(choice.E$Trial)
choice.E$Age.z<-as.vector(scale(choice.E$Age))
```

```
choice.E$Trial.z<-as.vector(scale(choice.E$Trial))
choice.E$TotalChoices_log <- log(choice.E$TotalChoices)


# model- - - - - - - - - - - -
full.Choice.E=glmer(Command.accomplished ~ Smell* (Age.z + Sex) + Trial.z +
                  offset(TotalChoices_log) +
                  (1+Smell.Smell2+Trial.z|AnimalID),
                family=binomial,
                data=choice.E,
                glmerControl(optimizer="bobyqa", optCtrl=list(maxfun=2e5)))
summary(full.Choice.E)

#check assumptions - - - - - - - - - - -
#Collinearity
full.Choice.E.m.coll=lme4::lmer(Command.accomplished ~ Smell + (Age.z + Sex) + Trial.z +
                            (1+Smell.Smell2+Trial.z||AnimalID),
                          data=choice.E)
round(vif(full.Choice.E.m.coll), 3) # fine if below 3
#Model stability
bin.stab.E <- glmm.model.stab(model.res = full.Choice.E)
bin.stab.E$summary[, -1]
m.stab.plot(bin.stab.E$summary[, -1]) # good

#Null-Full model comparison  - - - - - - - - - - - - - - - -
null.Choice.E=glmer(Command.accomplished ~ Trial.z +
                  offset(TotalChoices_log) +
                  (1+Smell.Smell2+Trial.z||AnimalID),
                family=binomial,
                data=choice.E,
                glmerControl(optimizer="bobyqa", optCtrl=list(maxfun=2e5)))

summary(null.Choice.E)

anova_full.Choice.E <- anova(null.Choice.E,full.Choice.E)
print(anova_full.Choice.E) # ns

# Confidence intervals
boot.bin.Choice.E=boot.lmer(m=full.Choice.E, discard.warnings=F, nboots=100, para=F,
                      resol=1000, level=0.95, use=c("task", "age"))
boot.bin.Choice.E$ci.estimates
m.stab.plot(boot.bin.Choice.E$ci.estimates) #not pretty -> small sample size



#________________________#
## * First Choice         ----
#________________________#

# Reduce database to command accomplished on a certain side
FiChoice.E <- combined_table.E %>%
  filter(TrialNo == "Test1") %>%
  mutate(Command.accomplished = ifelse(Side.Smell2 == "", NA, Command.accomplished ))
```

```
FiChoice.E <- FiChoice.E %>%  filter(Command.accomplished != "NA")

# check for random slopes - - - - - - - - - - -
xx.fe.re=fe.re.tab(fe.model="Command.accomplished ~ Smell * (Age + Sex) + Trial",
          re="(1|AnimalID)", data=FiChoice.E)
summary(xx.fe.re)

#factors are already dummycoded in fe.re. function
FiChoice.E=xx.fe.re$data #place in new object

# z-transform numeric predictors
FiChoice.E$Age.z<-as.vector(scale(FiChoice.E$Age))

# model- - - - - - - - - - - -
full.FiChoice.E=glmer(Command.accomplished ~ Smell*(Age.z + Sex) +
             (1+Smell.Smell2||AnimalID),
            family=binomial,
            data=FiChoice.E,
            glmerControl(optimizer="bobyqa", optCtrl=list(maxfun=2e5)))
summary(full.FiChoice.E)

#check assumptions - - - - - - - - - - -
#Collinearity
full.FiChoice.E.m.coll=lme4::lmer(Command.accomplished ~ Smell + Age.z + Sex +
                  (1+Smell.Smell2||AnimalID),
                 data=FiChoice.E)
round(vif(full.FiChoice.E.m.coll), 3) # fine if below 3
#Model stability
bin.stab.fc.E <- glmm.model.stab(model.res = full.FiChoice.E)
bin.stab.fc.E$summary[, -1]
m.stab.plot(bin.stab.fc.E$summary[, -1]) # good


#Null-Full model comparison  - - - - - - - - - - - - - - - -
null.FiChoice.E=glmer(Command.accomplished ~ 1 +
             (1+Smell.Smell2||AnimalID),
            family=binomial,
            data=FiChoice.E,
            glmerControl(optimizer="bobyqa", optCtrl=list(maxfun=2e5)))

summary(null.FiChoice.E)

anova_full.FiChoice.E <- anova(null.FiChoice.E,full.FiChoice.E)
print(anova_full.FiChoice.E) # ns

# Confidence intervals
boot.bin.FiChoice.E=boot.lmer(m=full.FiChoice.E, discard.warnings=F, nboots=100, para=F,
               resol=1000, level=0.95, use=c("task", "age"))
boot.bin.FiChoice.E$ci.estimates
m.stab.plot(boot.bin.FiChoice.E$ci.estimates)

confint(full.FiChoice.E, method = "boot", nsim = 100)
```

```
#________________________#
## * Proximity        ----
#________________________#

# Update the proximity column based on the condition in Side.Smell2
proximity.E <- combined_table.E %>%
  mutate(Proximity.to.target..G.T. = ifelse(Side.Smell2 == "", NA, Proximity.to.target..G.T. ))
proximity.E <- proximity.E %>%  filter(Proximity.to.target..G.T. != "0")
proximity.E <- proximity.E %>%  filter(Proximity.to.target..G.T. != "NA")
proximity.E <- proximity.E %>%  filter(TrialType == "Test")

# check for random slopes - - - - - - - - - - -
xx.fe.re=fe.re.tab(fe.model="Proximity.to.target..G.T. ~ Smell * (Age + Sex) + Trial",
          re="(1|AnimalID)", data=proximity.E)
summary(xx.fe.re)

#factors are already dummycoded in fe.re. function
proximity.E=xx.fe.re$data #place in new object

# z-transform numeric predictors
proximity.E$Trial<-as.numeric(proximity.E$Trial)
proximity.E$Age.z<-as.vector(scale(proximity.E$Age))
proximity.E$Trial.z<-as.vector(scale(proximity.E$Trial))

hist(log(proximity.E$Proximity.to.target..G.T.+1))
proximity.E$Proximity.to.target_log <- log(proximity.E$Proximity.to.target..G.T.+1)

# Model
full.proximity.E <- lmer(Proximity.to.target_log ~ Smell * (Age.z + Sex) + Trial.z +
                (1+Smell.Smell2+Trial.z|AnimalID),
               data = proximity.E)
summary(full.proximity.E)
summary(full.proximity.E)$varcor

#check assumptions - - - - - - - - - - -
sim_res <- simulateResiduals(fittedModel = full.proximity.E)
plot(sim_res)
ranef.diagn.plot(full.proximity.E) #BLUPS, all normally distributed, range small
#Collinearity (take out interactions)
full.ProxP.m.coll=lme4::lmer(Proximity.to.target_log  ~ Smell + Age.z + Sex + Trial.z +
                  (1+Smell.Smell2+Trial.z|AnimalID),
                 data=proximity.E)
round(vif(full.ProxP.m.coll), 3) # fine if below 2
#Model stability
full.stab.proximity.E=glmm.model.stab(model.res=full.proximity.E, contr=NULL,para=F, data=NULL)
m.stab.plot(full.stab.proximity.E$summary[, -1])  # for fixed effects


#Null-Full model comparison  - - - - - - - - - - - - - - -
null.proximity.E=lmer(Proximity.to.target_log ~ Trial.z +
              (1+Smell.Smell2+Trial.z|AnimalID),
```

```
                    data=proximity.E)

summary(null.proximity.E) #not converged

anova_full.proximity.E <- anova(null.proximity.E,full.proximity.E)
print(anova_full.proximity.E) # ns

#confidence intervals
boot.full.proximity.E=boot.lmer(m=full.proximity.E, discard.warnings=F,
                             nboots=100, para=T, n.cores=6, resol=100, level=0.95)
round(boot.full.proximity.E$ci.estimates, 3) #extract them
m.stab.plot(boot.full.proximity.E$ci.estimates)

confint(full.proximity.E, method = "boot", nsim = 100)

#________________________#
## *  Sniffing target       ----
#________________________#

# Update the sniffing column based on the condition in Side.Smell2
sniffingTP.E <- combined_table.E %>%
  mutate(Sniffing.target.PropP = ifelse(Side.Smell2 == "", NA, Sniffing.target.PropP ))
sniffingTP.E <- sniffingTP.E %>%  filter(Sniffing.target.PropP != "NA")

# check for random slopes - - - - - - - - - - -
xx.fe.re=fe.re.tab(fe.model="Sniffing.target.PropP ~ Smell * (Age + Sex) + Trial",
                re="(1|AnimalID)", data=sniffingTP.E)
summary(xx.fe.re)

#factors are already dummycoded in fe.re. function
sniffingTP.E=xx.fe.re$data #place in new object

# z-transform numeric predictors
sniffingTP.E$Trial<-as.numeric(sniffingTP.E$Trial)
sniffingTP.E$Age.z<-as.vector(scale(sniffingTP.E$Age))
sniffingTP.E$Trial.z<-as.vector(scale(sniffingTP.E$Trial))

#transform because beta cannot handle 0 and 1!!!
sniffingTP.E$Sniffing.target.PropP=beta.tr(sniffingTP.E$Sniffing.target.PropP)
sniffingTP.E$Sniffing.target.PropP  <- ifelse(sniffingTP.E$Sniffing.target.PropP  > 1, 0.9999,
sniffingTP.E$Sniffing.target.PropP)

hist((sniffingTP.E$Sniffing.target.PropP)) #it's a proportion, they can look weird. Just has to be bound
beweeen 0 and 1

# Model
full.sniffingTP.E <- glmmTMB(Sniffing.target.PropP ~ Smell * (Age.z + Sex) + Trial.z +
                           (1+Smell.Smell2+Trial.z||AnimalID),
                          family = beta_family,
                          data = sniffingTP.E)

summary(full.sniffingTP.E)
summary(full.sniffingTP.E)$varcor
```

```
#check assumptions - - - - - - - - - - -
ranef.diagn.plot(full.sniffingTP.E) #BLUPS, all normally distributed, range small
#Collinearity (take out interactions)
full.SniffTP.E.m.coll=lme4::lmer(Sniffing.target.PropP ~ Smell + Age.z + Sex + Trial.z +
                               (1+Smell.Smell2+Trial.z||AnimalID),
                             data=sniffingTP.E)
round(vif(full.SniffTP.E.m.coll), 3) # fine if below 2
#Model stability
stab.full.SniffTP.E=glmmTMB.stab(model.res=full.sniffingTP.E, para=T,
                             data=sniffingTP.E)
table(stab.full.SniffTP.E$detailed$converged)
m.stab.plot(stab.full.SniffTP.E$summary[, -1]) #good

#Null-Full model comparison  - - - - - - - - - - - - - - - -
null.SniffTP.E=glmmTMB(Sniffing.target.PropP ~ Trial.z +
                      (1+Smell.Smell2+Trial.z||AnimalID),
                    family = beta_family,
                    data = sniffingTP.E)

summary(null.SniffTP.E) #converged

anova_full.SniffTP.E <- anova(null.SniffTP.E,full.sniffingTP.E)
print(anova_full.SniffTP.E) # not significant

#confidence intervals
boot.full.sniffingTP.E=boot.glmmtmb(m=full.sniffingTP.E, data=sniffingTP.E,
                               discard.non.conv=F, nboots=1000, para=T, resol=100,
                               level=0.95, n.cores="all-1") #all-1 leaves you capacity on teh laptop to do other
things

boot.full.sniffingTP.E$ci.estimates$fe
m.stab.plot(boot.full.sniffingTP.E$ci.estimates$fe, 3)

# HERE ----
confint(full.sniffingTP.E, method = "wald")

#plot Age effect
ggplot(sniffingTP.E,aes(Age.z, Sniffing.target.PropP)) +
  stat_summary(fun.data=mean_cl_normal) +
  geom_smooth(method='lm', formula= y~x)

#________________________#
## * Engaging target       ----
#________________________#

# Update the column based on the condition in Side.Smell2
EngagingTP.E <- combined_table.E %>%
  mutate(Engaging.with.target.PropP = ifelse(Side.Smell2 == "", NA, Engaging.with.target.PropP ))
EngagingTP.E <- EngagingTP.E %>%  filter(Engaging.with.target.PropP != "NA")
EngagingTP.E <- EngagingTP.E %>%  filter(Engaging.with.target.PropP != "0")

# check for random slopes - - - - - - - - - - -
```

```
xx.fe.re=fe.re.tab(fe.model="Engaging.with.target.PropP ~ Smell * (Age + Sex) + Trial",
               re="(1|AnimalID)", data=EngagingTP.E)
summary(xx.fe.re)

#factors are already dummycoded in fe.re. function
EngagingTP.E=xx.fe.re$data #place in new object

# z-transform numeric predictors
EngagingTP.E$Trial<-as.numeric(EngagingTP.E$Trial)
EngagingTP.E$Age.z<-as.vector(scale(EngagingTP.E$Age))
EngagingTP.E$Trial.z<-as.vector(scale(EngagingTP.E$Trial))

#transform because beta cannot handle 0 and 1!!!
EngagingTP.E$Engaging.with.target.PropP=beta.tr(EngagingTP.E$Engaging.with.target.PropP)
EngagingTP.E$Engaging.with.target.PropP  <- ifelse(EngagingTP.E$Engaging.with.target.PropP  > 1,
0.9999, EngagingTP.E$Engaging.with.target.PropP)

hist((EngagingTP.E$Engaging.with.target.PropP))

# Model
full.EngagingTP.E <- glmmTMB(Engaging.with.target.PropP ~ Smell * (Age.z + Sex) + Trial.z +
                          (1+Smell.Smell2+Trial.z||AnimalID),
                         family = beta_family,
                         data = EngagingTP.E)

summary(full.EngagingTP.E)
summary(full.EngagingTP.E)$varcor

#check assumptions - - - - - - - - - - -
ranef.diagn.plot(full.EngagingTP.E) #BLUPS, all normally distributed, range small
#Collinearity (take out interactions)
full.EngagTP.E.m.coll=lme4::lmer(Engaging.with.target.PropP ~ Smell + Age.z + Sex + Trial.z +
                            (1+Smell.Smell2+Trial.z||AnimalID),
                           data=EngagingTP.E)
round(vif(full.EngagTP.E.m.coll), 3) # fine if below 2
#Model stability
stab.full.EngagTP.E=glmmTMB.stab(model.res=full.EngagingTP.E, para=T,
                           data=EngagingTP.E)
table(stab.full.EngagTP.E$detailed$converged)
m.stab.plot(stab.full.EngagTP.E$summary[, -1]) #good

#Null-Full model comparison  - - - - - - - - - - - - - - - -
null.EngagTP.E=glmmTMB(Engaging.with.target.PropP ~ Trial.z +
                    (1+Smell.Smell2+Trial.z||AnimalID),
                  family = beta_family,
                  data = EngagingTP.E)

summary(null.EngagTP.E) #converged

anova_full.EngagTP.E <- anova(null.EngagTP.E,full.EngagingTP.E)
print(anova_full.EngagTP.E) # not significant

#confidence intervals
```

```
boot.full.EngagingTP.E=boot.glmmtmb(m=full.EngagingTP.E, data=EngagingTP.E,
                        discard.non.conv=F, nboots=1000, para=T, resol=100,
                        level=0.95, n.cores="all-1") #all-1 leaves you capacity on teh laptop to do other
things

boot.full.EngagingTP.E$ci.estimates$fe
m.stab.plot(boot.full.EngagingTP.E$ci.estimates$fe, 3)

confint(full.EngagingTP.E, method = "wald")


#________________________#
## * Looking at target       ----
#________________________#

# Update the column based on the condition in Side.Smell2
LookingTP.E <- combined_table.E %>%
  mutate(Looking.at.target.GT.Prop = ifelse(Side.Smell2 == "", NA, Looking.at.target.GT.Prop))

LookingTP.E <- LookingTP.E %>%  filter(Looking.at.target.GT.Prop != "NA")
LookingTP.E <- LookingTP.E %>%  filter(Looking.at.target.GT.Prop != "0")

# check for random slopes - - - - - - - - - - -
xx.fe.re=fe.re.tab(fe.model="Looking.at.target.GT.Prop ~ Smell * Age + Sex + Trial",
            re="(1|AnimalID)", data=LookingTP.E)
summary(xx.fe.re)

#factors are already dummycoded in fe.re. function
LookingTP.E=xx.fe.re$data #place in new object

# z-transform numeric predictors
LookingTP.E$Trial<-as.numeric(LookingTP.E$Trial)
LookingTP.E$Age.z<-as.vector(scale(LookingTP.E$Age))
LookingTP.E$Trial.z<-as.vector(scale(LookingTP.E$Trial))

#transform because beta cannot handle 0 and 1!!!
LookingTP.E$Looking.at.target.GT.Prop=beta.tr(LookingTP.E$Looking.at.target.GT.Prop)
LookingTP.E$Looking.at.target.GT.Prop  <- ifelse(LookingTP.E$Looking.at.target.GT.Prop  > 1, 0.9999,
LookingTP.E$Looking.at.target.GT.Prop)

hist((LookingTP.E$Looking.at.target.GT.Prop))

# Model
full.LookingTP.E <- glmmTMB(Looking.at.target.GT.Prop ~ Smell * (Age.z + Sex) + Trial.z +
                     (1+Smell.Smell2+Trial.z||AnimalID),
                    family = beta_family,
                    data = LookingTP.E)

summary(full.LookingTP.E)
summary(full.LookingTP.E)$varcor

#check assumptions - - - - - - - - - - -
ranef.diagn.plot(full.LookingTP.E) #BLUPS, all normally distributed, range small
```

```
#Collinearity (take out interactions)
full.LookTP.E.m.coll=lme4::lmer(Looking.at.target.GT.Prop ~ Smell + Age.z + Sex + Trial.z +
                             (1+Smell.Smell2+Trial.z||AnimalID),
                           data=LookingTP.E)
round(vif(full.LookTP.E.m.coll), 3) # fine if below 2
#Model stability
stab.full.LookTP.E=glmmTMB.stab(model.res=full.LookingTP.E, para=T,data=LookingTP.E)
table(stab.full.LookTP.E$detailed$converged)
m.stab.plot(stab.full.LookTP.E$summary[, -1]) #good

#Null-Full model comparison - - - - - - - - - - - - - - - -
null.LookTP.E=glmmTMB(Looking.at.target.GT.Prop ~ Trial.z +
                    (1+Smell.Smell2+Trial.z||AnimalID),
                  family = beta_family,
                  data = LookingTP.E)

summary(null.LookTP.E) #converged

anova_full.LookTP.E <- anova(null.LookTP.E,full.LookingTP.E)
print(anova_full.LookTP.E) # not significant

#confidence intervals
boot.full.LookingTP.E=boot.glmmtmb(m=full.LookingTP.E, data=LookingTP.E,
                              discard.non.conv=F, nboots=1000, para=T, resol=100,
                              level=0.95, n.cores="all-1") #all-1 leaves you capacity on teh laptop to do other
things

boot.full.LookingTP.E$ci.estimates$fe
m.stab.plot(boot.full.LookingTP.E$ci.estimates$fe, 3)

confint(full.LookingTP.E, method = "wald")

#________________________#
## * Commands needed        ----
#________________________#

# Create the 'command.smell' column, where command number value is placed in rows where
Command.accomplished == 1
combined_table.E <- combined_table.E %>%
  group_by(TrialNo, AnimalID) %>%
  mutate(
    Command.Smell = ifelse(
      Command.accomplished == 1,
      first(Number.of.commands[!is.na(Number.of.commands)]),
      NA )
  ) %>%
  ungroup()

# Update the column based on the condition in Side.Smell2
Commands.E <- combined_table.E %>%
  mutate(Command.Smell = ifelse(Side.Smell2 == "", NA, Command.Smell))
Commands.E <- Commands.E %>%  filter(Command.Smell != "NA")
```

```
# check for random slopes - - - - - - - - - - -
xx.fe.re=fe.re.tab(fe.model="Command.Smell ~ Smell * (Age + Sex) + Trial",
          re="(1|AnimalID)", data=Commands.E)
summary(xx.fe.re)

#factors are already dummycoded in fe.re. function
Commands.E=xx.fe.re$data #place in new object

# z-transform numeric predictors
Commands.E$Trial<-as.numeric(Commands.E$Trial)
Commands.E$Age.z<-as.vector(scale(Commands.E$Age))
Commands.E$Trial.z<-as.vector(scale(Commands.E$Trial))

hist((Commands.E$Command.Smell))

# Model
full.Commands.E <- glmer(Command.Smell ~ Smell * (Age.z + Sex) + Trial.z +
                 (1+Smell.Smell2+Trial.z||AnimalID), #doesn't converge with random slopes
                family = poisson(),
                data = Commands.E)
summary(full.Commands.E)
summary(full.Commands.E)$varcor

#check assumptions - - - - - - - - - - -
overdisp.test(full.Commands.E) # not overdispersed (not sign.)
ranef.diagn.plot(full.Commands.E) #BLUPS, all normally distributed, range small
#Collinearity (take out interactions)
full.Command.m.coll=lme4::lmer(Command.Smell ~ Smell  + Age.z + Sex + Trial.z +
                    (1+Smell.Smell2+Trial.z||AnimalID),
                   data=Commands.E)
round(vif(full.Command.m.coll), 3) # fine if below 2
#Model stability
bin.stab=glmm.model.stab(model.res=full.Commands.E)
bin.stab$summary[, -1]
m.stab.plot(bin.stab$summary[, -1]) #ok

#Null-Full model comparison  - - - - - - - - - - - - - - - -
null.Commands.E <- glmer(Command.Smell ~ Trial.z +
                 (1+Smell.Smell2+Trial.z||AnimalID),
                family = poisson(),
                data = Commands.E)

summary(null.Commands.E) #converged

anova_full.Commands.E <- anova(null.Commands.E,full.Commands.E)
print(anova_full.Commands.E) # not significant


#confidence intervals
full.boot.Commands.E=boot.lmer(m=full.Commands.E,
                   nboots=100, para=F, n.cores=6, resol=100, level=0.95) #normally nboots=1000,
para=T but takes way longer
```

```
m.stab.plot(full.boot.Commands.E$ci.estimates)
full.boot.Commands.E$ci.estimates

confint(full.Commands.E, method = "Wald")


#________________________#
## * Latency       ----
#________________________#

# Update the column based on the condition in Side.Smell2
Latency.E <- combined_table.E %>%
  mutate(LatencySmell = ifelse(Side.Smell2 == "", NA, LatencySmell))
Latency.E <- Latency.E %>%  filter(LatencySmell != "NA",
                                   TrialType == "Test",
                                   Smell != "none")

hist((Latency.E$LatencySmell))
hist(log(Latency.E$LatencySmell+1))
Latency.E$LatencySmell_log <- log(Latency.E$LatencySmell+1)

# check for random slopes - - - - - - - - - - -
xx.fe.re=fe.re.tab(fe.model="LatencySmell_log ~ Smell * (Age + Sex) + Trial",
                   re="(1|AnimalID)", data=Latency.E)
summary(xx.fe.re)

#factors are already dummycoded in fe.re. function
Latency.E=xx.fe.re$data #place in new object

# z-transform numeric predictors
Latency.E$Trial<-as.numeric(Latency.E$Trial)
Latency.E$Age.z<-as.vector(scale(Latency.E$Age))
Latency.E$Trial.z<-as.vector(scale(Latency.E$Trial))

# Model
full.lat.E <- lmer(LatencySmell_log ~ Smell* (Age.z + Sex) + Trial.z +
                     (1+Smell.Smell2+Trial.z||AnimalID),
                   data = Latency.E)

summary(full.lat.E)

#check assumptions - - - - - - - - - - -
sim_res <- simulateResiduals(fittedModel = full.lat.E)
testDispersion(sim_res) # if SQRT, not overdispersed (not sign.)
#Collinearity (take out interactions)
full.latD.E.m.coll=lmer(LatencySmell_log ~ Smell + Age.z + Sex + Trial.z +
                          (1+Smell.Smell2+Trial.z|AnimalID),
                        data = Latency.E)  # Body size and Species highly correlated
round(vif(full.latD.E.m.coll), 3) # fine if below 2
#Model stability
full.stab.E.lat=glmm.model.stab(model.res=full.lat, contr=NULL,para=F, data=NULL)
m.stab.plot(full.stab.E.lat$summary[-1])  # for fixed effects
```

```
#Null-Full model comparison - - - - - - - - - - - - - - - -
null.lat.E=lmer(LatencySmell_log ~  Age.z + Sex + Trial.z +
              (1+Smell.Smell2+Trial.z||AnimalID),
            data = Latency.E)

summary(null.lat.E) #converged

anova_full.lat.E <- anova(null.lat.E,full.lat.E)
print(anova_full.lat.E) # ns


# Confidence interval
boot.red.Latency.E=boot.lmer(m=red.lat, discard.warnings=F,
                        nboots=1000, para=T, n.cores=6, resol=1000, level=0.95)
round(boot.red.Latency.E$ci.estimates, 3) #extract them
m.stab.plot(boot.red.Latency.E$ci.estimates)

boot.full.Latency.E=boot.lmer(m=full.lat, discard.warnings=F,
                         nboots=1000, para=T, n.cores=6, resol=1000, level=0.95)
m.stab.plot(boot.full.Latency.E$ci.estimates)
round(boot.full.Latency.E$ci.estimates, 3) #extract them

confint(red.lat, method = "Wald")


#-#-###-#-#-#-#-#-#-#-#-#-#-#-#-#-#-#-##-#-#
# 4. Models  -  Group differences    ----
#-#-###-#-#-#-#-#-#-#-#-#-#-#-#-#-#-#-##-#-#

#__________________________________#
## * Smelling environment     ----
#__________________________________#

# BETA MODEL DID NOT CONVERGE

#-#-#-#-#-#-#-#-#-#-#--#-#-#-#- BINARY MODEL

#change to yes/no
SniffEP.I <- combined_table %>%
  group_by(AnimalID, Group, TrialNo, TrialType, Sex, Trial, Age) %>% # Group by AnimalID and
TrialNO, along with other columns
  summarise(Sniffing.environment.Prop_Sum = sum(Sniffing.environment.Prop, na.rm = TRUE),
.groups = "drop") # Sum Sniffing.environment.Prop and remove grouping

SniffEP.I <- SniffEP.I %>%  filter(TrialType == "Test")

# check for random slopes - - - - - - - - - - -
xx.fe.re=fe.re.tab(fe.model="Sniffing.environment.Prop_Sum ~ Group *(Age + Sex) + Trial",
              re="(1|AnimalID)", data=SniffEP.I)

#factors are already dummycoded in fe.re. function
SniffEP.I=xx.fe.re$data #place in new object
```

```
SniffEP.I$Trial<-as.numeric(SniffEP.I$Trial)
SniffEP.I$Age.z<-as.vector(scale(SniffEP.I$Age))
SniffEP.I$Trial.z<-as.vector(scale(SniffEP.I$Trial))

SniffEP.I <- SniffEP.I %>%
  mutate(Sniffing.environment.Binary = ifelse(Sniffing.environment.Prop_Sum != 0, 1, 0))

# model- - - - - - - - - - - -
full.Sniff2.I=glmer(Sniffing.environment.Binary ~ Group *(Age.z + Sex) + Trial.z +
                  (1+Trial.z|AnimalID),
                family=binomial, data=SniffEP.I,
                glmerControl(optimizer="bobyqa", optCtrl=list(maxfun=2e5)))

summary(full.Sniff2.I) #converges
summary(full.Sniff2.I)$varcor

#check assumptions - - - - - - - - - - -
#Collinearity (take out interactions)
full.Sniff2.I.m.coll=lme4::lmer(Sniffing.environment.Binary ~ Group+(Age.z + Sex) + Trial.z +
                            (1+Trial.z||AnimalID),
                          data=SniffEP.I)
round(vif(full.Sniff2.I.m.coll), 3) # fine if below 3
#Model stability
bin.stab=glmm.model.stab(model.res=full.Sniff2.I)
bin.stab$summary[, -1]
m.stab.plot(bin.stab$summary[, -1]) #fine

#Null-Full model comparison  - - - - - - - - - - - - - - - -
null.Sniff2.I=glmer(Sniffing.environment.Binary ~ Trial.z +
                  (1+Trial.z|AnimalID),
                family=binomial, data=SniffEP.I,
                glmerControl(optimizer="bobyqa", optCtrl=list(maxfun=2e5)))

summary(null.Sniff2.I) #converged

anova_full.Sniff2.I <- anova(null.Sniff2.I,full.Sniff2.I)
print(anova_full.Sniff2.I) # ns


# Confidence intervals
boot.bin.Sniff2.I=boot.lmer(m=full.Sniff2.I, discard.warnings=F, nboots=100, para=F,
                       resol=100, level=0.95, use=c("task", "age"))
boot.bin.Sniff2.I$ci.estimates
m.stab.plot(boot.bin.Sniff2.I$ci.estimates) #not pretty -> small sample size

confint(full.Sniff2.I, method = "Wald")

#________________________________#
## * Looking at experimenter ----
#________________________________#

# Sum sniffing enviroment
LookEPG.I <- combined_table %>%
```

```
  group_by(AnimalID, Group, TrialNo, TrialType, Sex, Trial, Age) %>% # Group by AnimalID and
TrialNO, along with other columns
  summarise(Looking.at.experimenter.GT.Prop_Sum = sum(Looking.at.experimenter.GT.Prop, na.rm =
TRUE), .groups = "drop") # Sum Sniffing.environment.Prop and remove grouping

LookEPG.I <- LookEPG.I %>%  filter(TrialType == "Test")

# check for random slopes - - - - - - - - - - -
xx.fe.re=fe.re.tab(fe.model="Looking.at.experimenter.GT.Prop_Sum ~ Group*(Age + Sex) + Trial",
            re="(1|AnimalID)", data=LookEPG.I)

#factors are already dummycoded in fe.re. function
LookEPG.I=xx.fe.re$data #place in new object

# z-transform numeric predictors
LookEPG.I$Trial<-as.numeric(LookEPG.I$Trial)
LookEPG.I$Age.z<-as.vector(scale(LookEPG.I$Age))
LookEPG.I$Trial.z<-as.vector(scale(LookEPG.I$Trial))

#transform because beta cannot handle 0 and 1!!!
LookEPG.I$Looking.at.experimenter.GT.Prop_Sum=beta.tr(LookEPG.I$Looking.at.experimenter.GT.Pr
op_Sum)
LookEPG.I$Looking.at.experimenter.GT.Prop_Sum  <-
ifelse(LookEPG.I$Looking.at.experimenter.GT.Prop_Sum  > 1, 0.9999,
LookEPG.I$Looking.at.experimenter.GT.Prop_Sum)

hist((LookEPG.I$Looking.at.experimenter.GT.Prop_Sum))

# Model
full.LookEPG.I <- lmer(Looking.at.experimenter.GT.Prop_Sum ~ Group *(Age.z + Sex) + Trial.z +
                 (1+Trial.z||AnimalID),
               data = LookEPG.I)

summary(full.LookEPG.I)
summary(full.LookEPG.I)$varcor

#check assumptions - - - - - - - - - - -
ranef.diagn.plot(full.LookEPG.I) #BLUPS, all normally distributed, range small
#Collinearity (take out interactions)
full.LookEPG.I.m.coll=lme4::lmer(Looking.at.experimenter.GT.Prop_Sum  ~ Group + Age.z + Sex +
Trial.z +
                      (1+Trial.z||AnimalID),
                    data=LookEPG.I)
round(vif(full.LookEPG.I.m.coll), 3) # fine if below 2
#Model stability
stab.full.EngagTP=glmmTMB.stab(model.res=full.LookEPG.I, para=T, data=LookEPG.I)
table(stab.full.LookEPG.I$detailed$converged)
m.stab.plot(stab.full.LookEPG.I$summary[, -1])


# Null-Full model comparison
null.LookEPG.I <- lmer(Looking.at.experimenter.GT.Prop_Sum ~ Trial.z +
                 (1+Trial.z||AnimalID),
```

```
                    data = LookEPG.I)

summary(null.LookEPG.I) # Check null model summary
anova_full_LookEPG.I <- anova(null.LookEPG.I, full.LookEPG.I)
print(anova_full_LookEPG.I) #not significant


# Confidence interval
boot.full.LookEPG.I=boot.lmer(m=full.LookEPG.I, discard.warnings=F,
                         nboots=100, para=T, n.cores=6, resol=1000, level=0.95)
round(boot.full.LookEPG.I$ci.estimates, 3) #extract them
m.stab.plot(boot.full.LookEPG.I$ci.estimates)

confint(full.LookEPG.I, method = "Wald")

#________________________________#
## * Proximity experimenter ----
#________________________________#

# Sum prox experimenter
ProxEP.I <- combined_table %>%
  group_by(AnimalID, Group, Sex, Trial.duration, Trial, Age) %>% # Group by AnimalID and TrialNO,
along with other columns
  summarise(Proximity.experimenter.Prop_Sum = sum(Proximity.experimenter.Prop, na.rm = TRUE),
.groups = "drop") # Sum Sniffing.environment.Prop_Sum and remove grouping

# check for random slopes - - - - - - - - - - -
xx.fe.re=fe.re.tab(fe.model="Proximity.experimenter.Prop_Sum ~ Group* (Age + Sex) + Trial",
                re="(1|AnimalID)", data=ProxEP.I)

#factors are already dummycoded in fe.re. function
ProxEP.I=xx.fe.re$data #place in new object

# z-transform numeric predictors
ProxEP.I$Trial<-as.numeric(ProxEP.I$Trial)
ProxEP.I$Age.z<-as.vector(scale(ProxEP.I$Age))
ProxEP.I$Trial.z<-as.vector(scale(ProxEP.I$Trial))

#transform because beta cannot handle 0 and 1!!!
ProxEP.I$Proximity.experimenter.Prop_Sum=beta.tr(ProxEP.I$Proximity.experimenter.Prop_Sum)
ProxEP.I$Proximity.experimenter.Prop_Sum  <- ifelse(ProxEP.I$Proximity.experimenter.Prop_Sum  >
1, 0.9999, ProxEP.I$Proximity.experimenter.Prop_Sum)

# Model
full.ProxEP.I <- glmmTMB(Proximity.experimenter.Prop_Sum ~ Group*(Age.z + Sex) + Trial.z +
                     (1+Trial.z||AnimalID),
                    family = beta_family(),
                    data = ProxEP.I)

summary(full.ProxEP.I)
summary(full.ProxEP.I)$varcor

#check assumptions - - - - - - - - - - -
```

```
ranef.diagn.plot(full.ProxEP.I) #BLUPS, all normally distributed, range small
#Collinearity (take out interactions)
full.ProxEP.I.m.coll=lme4::lmer(Proximity.experimenter.Prop_Sum  ~ Group + Age.z + Sex + Trial.z +
                              (1+Trial.z||AnimalID),
                            data=ProxEP.I)
round(vif(full.ProxEP.I.m.coll), 3) # fine if below 2
#Model stability
stab.full.ProxP=glmmTMB.stab(model.res=full.ProxEP.I, para=T,data=ProxEP.I)
table(stab.full.ProxEP.I$detailed$converged)
m.stab.plot(stab.full.ProxEP.I$summary[, -1])

# Null-Full model comparison
null.ProxEP.I <- glmmTMB(Proximity.experimenter.Prop_Sum ~ Trial.z +
                           (1+Trial.z||AnimalID),
                         family = beta_family(),
                         data = ProxEP.I)

drop1(full.ProxEP.I, test="Chisq")
# Group sign

# reduced model
# Model
red.ProxEP.I <- glmmTMB(Proximity.experimenter.Prop_Sum ~ Group+(Age.z + Sex) + Trial.z +
                          (1+Trial.z||AnimalID),
                        family = beta_family(),
                        data = ProxEP.I)

summary(red.ProxEP.I)

anova_red_ProxEP.I <- anova(null.ProxEP.I, red.ProxEP.I)
print(anova_red_ProxEP.I) #ns but Group significant

emmeans(red.ProxEP.I, pairwise ~ Group)
# Control < Test (0.012)

#confidence intervals
boot_fun <- function(model) {
  as.numeric(fixef(model)$cond)  # Extract conditional fixed effects and coerce to numeric
}
# Perform bootstrapping
#set.seed(123)  # For reproducibility
boot_res_ProxEP.I <- bootMer(red.ProxEP.I, boot_fun, nsim = 100)  # Adjust nsim for better precision
# Calculate 95% confidence intervals
boot_ci_ProxEP.I <- apply(boot_res_ProxEP.I$t, 2, quantile, probs = c(0.025, 0.975))
# Display the confidence intervals
print(boot_ci_Prox_EP)

confint(red.ProxEP.I, method = "Wald")

#_________________________________#
## * Higher Tail wag ----
#_________________________________#
```

```
# Sum prox experimenter
TailHP.I <- combined_table %>%
  group_by(AnimalID, Group, Sex, Trial, Age) %>% # Group by AnimalID and TrialNO, along with other
columns
  summarise(Tail.wagging.high.Prop_sum = sum(Tail.wagging.high.Prop, na.rm = TRUE), .groups =
"drop") # Sum Sniffing.environment.Prop and remove grouping

# check for random slopes - - - - - - - - - - -
xx.fe.re=fe.re.tab(fe.model="Tail.wagging.high.Prop_sum ~ Group*(Age + Sex) + Trial",
           re="(1|AnimalID)", data=TailHP.I)

#factors are already dummycoded in fe.re. function
TailHP.I=xx.fe.re$data #place in new object

# z-transform numeric predictors
TailHP.I$Trial<-as.numeric(TailHP.I$Trial)
TailHP.I$Age.z<-as.vector(scale(TailHP.I$Age))
TailHP.I$Trial.z<-as.vector(scale(TailHP.I$Trial))

#transform because beta cannot handle 0 and 1!!!
TailHP.I$Tail.wagging.high.Prop_sum=beta.tr(TailHP.I$Tail.wagging.high.Prop_sum)
TailHP.I$Tail.wagging.high.Prop_sum  <- ifelse(TailHP.I$Tail.wagging.high.Prop_sum  > 1, 0.9999,
TailHP.I$Tail.wagging.high.Prop_sum)

# Model
full.TailHP.I <- glmmTMB(Tail.wagging.high.Prop_sum ~ Group*(Age.z + Sex) + Trial.z +
                  (1+Trial.z||AnimalID), #trial doesn't converge
                 family = beta_family(),
                 data = TailHP.I)

summary(full.TailHP.I)
summary(full.TailHP.I)$varcor


#check assumptions - - - - - - - - - - -
ranef.diagn.plot(full.TailHP.I) #BLUPS, all normally distributed, range small
#Collinearity (take out interactions)
full.TailHP.I.m.coll=lme4::lmer(Tail.wagging.high.Prop_sum ~ Group + Age.z + Sex + Trial.z +
                        (1+Trial.z||AnimalID),
                       data=TailHP.I)
round(vif(full.TailHP.I.m.coll), 3) # fine if below 2
#Model stability
stab.full.TailHP.I=glmmTMB.stab(model.res=full.TailHP.I, para=T, data=TailHP.I)
table(stab.full.TailHP.I$detailed$converged)
m.stab.plot(stab.full.TailHP.I$summary[, -1])

# Null-Full model comparison
null.TailHP.I <- glmmTMB(Tail.wagging.high.Prop_sum ~ Trial.z +
                  (1+Trial.z||AnimalID),
                 family = beta_family(),
                 data = TailHP.I)

summary(null.TailHP.I) # Check null model summary
```

```
anova_full_TailHP.I <- anova(null.TailHP.I, full.TailHP.I)
print(anova_full_TailHP.I) #not significant


#confidence intervals
#set.seed(123)  # For reproducibility
boot_res_TailHP.I <- bootMer(full.TailHP.I, boot_fun, nsim = 1000)  # Adjust nsim for better precision
# Calculate 95% confidence intervals
boot_ci_TailHP.I <- apply(boot_res_TailHP.I$t, 2, quantile, probs = c(0.025, 0.975))
# Display the confidence intervals
print(boot_ci)

boot_funX <- function(data, indices) {
  # Resample data
  sampled_data <- data[indices, ]

  # Refit the model
  model <- tryCatch(
    update(full.TailHP.I, data = sampled_data),
    error = function(e) return(rep(NA, length(fixef(full.TailHP.I))))
  )

  # Check if model fit was successful
  if (inherits(model, "glmmTMB")) {
    stat <- fixef(model)$cond  # Extract only fixed effects
  } else {
    stat <- rep(NA, length(fixef(full.TailHP.I)$cond))  # Return NA if model fitting fails
  }

  # Convert to a numeric vector (remove names if necessary)
  return(as.numeric(stat))
}

boot_res_TailHP.I <- boot(data = model.frame(full.TailHP.I), statistic = boot_funX, R = 100)
boot_res_TailHP.I$ci

confint(full.TailHP.I, method = "Wald")


#_________________________________#
## * Lower Tail wag ----
#_________________________________#

# BETA didn't converge

# Binary model #

TailLP2.I <- combined_table %>%
  group_by(AnimalID, Group, TrialNo, TrialType, Sex, Trial, Age) %>% # Group by AnimalID and
TrialNO, along with other columns
  summarise(Tail.wagging.low.Prop_sum = sum(Tail.wagging.low.Prop, na.rm = TRUE), .groups =
"drop") # Sum Sniffing.environment.Prop and remove grouping
```

```
TailLP2.I <- TailLP2.I %>%
  mutate(Tail.wagging.low.Binary = ifelse(Tail.wagging.low.Prop_sum != 0, 1, 0))

# check for random slopes - - - - - - - - - - -
xx.fe.re=fe.re.tab(fe.model="Tail.wagging.low.Binary ~ Group* (Age + Sex) + Trial",
           re="(1|AnimalID)", data=TailLP2.I)

#factors are already dummycoded in fe.re. function
TailLP2.I=xx.fe.re$data #place in new object

# z-transform numeric predictors
TailLP2.I$Trial<-as.numeric(TailLP2.I$Trial)
TailLP2.I$Age.z<-as.vector(scale(TailLP2.I$Age))
TailLP2.I$Trial.z<-as.vector(scale(TailLP2.I$Trial))

# model- - - - - - - - - - - -
full.TailLP2.I=glmer(Tail.wagging.low.Binary ~ Group* (Age.z + Sex) + Trial.z +
              (1+Trial.z||AnimalID),
             family=binomial, data=TailLP2.I)

summary(full.TailLP2.I) #converges
summary(full.TailLP2.I)$varcor

#check assumptions - - - - - - - - - - -
#Collinearity (take out interactions)
full.TailLP2.I.m.coll=lme4::lmer(Tail.wagging.low.Binary ~ Group + Age.z + Sex + Trial.z +
                   (Trial.z||AnimalID),
                  data=TailLP2.I)
round(vif(full.TailLP2.I.m.coll), 3) # fine if below 3
#Model stability
bin.stab=glmm.model.stab(model.res=full.TailLP2.I)
bin.stab$summary[, -1]
m.stab.plot(bin.stab$summary[, -1]) #fine


#Null-Full model comparison  - - - - - - - - - - - - - - - -
null.TailLP2.I=glmer(Tail.wagging.low.Binary ~ Trial.z +
              (1+Trial.z||AnimalID),
             family=binomial, data=TailLP2.I)

summary(null.TailLP2.I) #converged

anova_full.TailLP2.I <- anova(null.TailLP2.I,full.TailLP2.I)
print(anova_full.TailLP2.I) # significant

#Test for each predictor  - - - - - - - - - - - - - - - -
drop1(full.TailLP2.I, test="Chisq")
# Group significant

emmeans(full.TailLP2.I, pairwise ~ Group*Sex)
# ns

#reduced model
```

```
red.TailLP2.I=glmer(Tail.wagging.low.Binary ~ Group+ (Age.z + Sex) + Trial.z +
                (1+Trial.z||AnimalID),
               family=binomial, data=TailLP2.I)

summary(red.TailLP2.I)

emmeans(red.TailLP2.I, pairwise~ Group)

# Confidence intervals
boot.bin.TailLP2.I=boot.lmer(m=full.TailLP2.I, discard.warnings=F, nboots=100, para=F,
                     resol=100, level=0.95, use=c("task", "age"))
boot.bin.TailLP2.I$ci.estimates
m.stab.plot(boot.bin.TailLP2.I$ci.estimates) #not pretty -> small sample size

confint(red.TailLP2.I, method = "Wald")

#__________________________________#
## * Not accomplished            ----
#__________________________________#

# Reduce rows
NotAcc.I <- combined_table %>%
  group_by(AnimalID, Group, Sex, Age) %>% # Group by AnimalID and TrialNO, along with other
columns
  summarise(Not.accomplished = sum((Not.accomplished), na.rm = TRUE), .groups = "drop")

NotAcc.I <- NotAcc.I %>%  filter(Not.accomplished != "NaN")

NotAcc.I$Age.z<-as.vector(scale((NotAcc.I$Age)))

#Create the model - - - - - - - - - - -
full.NotAcc.I=glmer(Not.accomplished ~ Group *(Age.z + Sex)
               + (1|AnimalID),
               family=poisson,
               glmerControl(optimizer="bobyqa", optCtrl=list(maxfun=2e5)),
               data=NotAcc.I)

summary(full.NotAcc.I)

# Perform the overdispersion check using simulateResiduals from the DHARMa package
sim_res <- simulateResiduals(full.NotAcc.I)
plot(sim_res)  # plot the residuals to check for overdispersion
testOverdispersion(sim_res)  # test for overdispersion

# Random effects diagnostics - checking BLUPs (Best Linear Unbiased Predictors)
ranef_diagn_plot <- ranef(full.NotAcc.I)  # Extract random effects (BLUPs)
plot(ranef_diagn_plot)  # Check for normal distribution of random effects

# VIF check for multicollinearity - exclude interaction terms
full.NotAcc.I_coll <- lm(Not.accomplished ~ Group + Age.z + Sex,
                   data = NotAcc.I)
vif(full.NotAcc.I_coll)  # Variance Inflation Factor to check for collinearity
```

```
# Simulate residuals for model stability
simres <- simulateResiduals(full.NotAcc.I)
plot(simres)  # Plot the simulated residuals

#Null-Full model comparison
null.NotAcc.I <- glmer(Not.accomplished ~ 1 +
                    (1|AnimalID),
                  family = poisson,
                  data = NotAcc.I)   #doesn't converge but full summary ns
summary(null.NotAcc.I)  # Summary of the null model

anova_full_NotAcc.I <- anova(null.NotAcc.I, full.NotAcc.I)
print(anova_full_NotAcc.I)  # not significant

table(combined_table$Not.accomplished,combined_table$Group)
#8/189=0.04
#29/345=0.08


#-#-#-#-#-#-#-#-#-#--#-#-#-#- BINARY MODEL

#change to yes/no
NotAcc2.I <- combined_table %>%
  group_by(AnimalID, Group, Sex, Age) %>% # Group by AnimalID and TrialNO, along with other
columns
  summarise(Not.accomplished_Bin = sum(Not.accomplished, na.rm = TRUE), .groups = "drop") # Sum
Sniffing.environment.Prop and remove grouping

NotAcc2.I$Age.z<-as.vector(scale(NotAcc2.I$Age))

NotAcc2.I <- NotAcc2.I %>%
  mutate(Not.accomplished_Bin = ifelse(Not.accomplished_Bin != 0, 1, 0))

# model- - - - - - - - - - - -
full.NotAcc2.I=glm(Not.accomplished_Bin ~ Group *(Age.z + Sex),
               family=binomial, data=NotAcc2.I)

summary(full.NotAcc2.I) #converges
summary(full.NotAcc2.I)$varcor

#check assumptions - - - - - - - - - - -
#Collinearity (take out interactions)
full.NotAcc2.I.m.coll=lm(Not.accomplished_Bin ~ Group + Age.z + Sex,
                    data=NotAcc2.I)
round(vif(full.NotAcc2.I.m.coll), 3) # fine if below 3
#Model stability
bin.stab=glmm.model.stab(model.res=full.Sniff2)
bin.stab$summary[, -1]
m.stab.plot(bin.stab$summary[, -1]) #fine

#Null-Full model comparison  - - - - - - - - - - - - - - - -
null.NotAcc2.I=glm(Not.accomplished_Bin ~ 1,
               family=binomial, data=NotAcc2.I)
```

```
summary(null.NotAcc2.I) #converged

anova_full.NotAcc2.I <- anova(null.NotAcc2.I,full.NotAcc2.I)
print(anova_full.NotAcc2.I) # no significant

# Confidence intervals
boot.bin.NotAcc2.I=boot.lmer(m=full.NotAcc2.I, discard.warnings=F, nboots=100, para=F,
                     resol=100, level=0.95, use=c("task", "age"))
boot.bin.NotAcc2.I$ci.estimates
m.stab.plot(boot.bin.NotAcc2.I$ci.estimates)


#confidence intervals
full.boot.NotAcc=boot.lmer(m=full.NotAcc,
                    nboots=100, para=F, n.cores=6, resol=100, level=0.95) #normally nboots=1000,
para=T but takes way longer

m.stab.plot(full.boot.NotAcc$ci.estimates)
full.boot.NotAcc$ci.estimates

confint(full.NotAcc, method = "Wald")

#__________________________________#
## * Terminate (model)           ----
#__________________________________#

# Reduce rows
Abt <- combined_table %>%
  group_by(AnimalID, Group, Sex, Age) %>% # Group by AnimalID and TrialNO, along with other
columns
  summarise(Abort = sum((Abort), na.rm = TRUE), .groups = "drop")

Abt <- Abt %>%  filter(Abort != "NaN")

Abt$Age.z<-as.vector(scale((Abt$Age)))

#Create the model - - - - - - - - - - -
full.Abt=glmer(Abort ~ Group + Age.z + Sex
          + (1|AnimalID),
          family=poisson,
          data=Abt)

summary(full.Abt)

# Perform the overdispersion check using simulateResiduals from the DHARMa package
sim_res <- simulateResiduals(full.Abt)
plot(sim_res)  # plot the residuals to check for overdispersion
testOverdispersion(sim_res)  # test for overdispersion

# VIF check for multicollinearity - exclude interaction terms
full.Abt_coll <- lm(Abort ~ Group + Age.z + Sex,
              data = Abt)
```

```
vif(full.Abt_coll)  # Variance Inflation Factor to check for collinearity

# Simulate residuals for model stability
simres <- simulateResiduals(full.Abt)
plot(simres)  # Plot the simulated residuals

#Null-Full model comparison
null.Abt <- glmer(Abort ~ Age.z + Sex +
                  (1|AnimalID),
                family = poisson,
                data = Abt)   #doesn't converge but full summary ns
summary(null.Abt)  # Summary of the null model

anova_full_Abt <- anova(null.Abt, full.Abt)
print(anova_full_Abt)  # ns


#___________________________________#
## * Abort (Chisq)            ----
#___________________________________#
#just looking at the aborters
table(combined_table$Group, combined_table$Abort)
combined_table %>%
  group_by(Group) %>%
  summarise(
    total_animals = n_distinct(AnimalID)  # Count of unique animals per group
  )

# Create a 2x2 contingency table
aboInd <- matrix(c(10, 1, 31, 19), nrow = 2, byrow = TRUE) # Rows: Experimental, Control
colnames(aboInd) <- c("Experimental", "Control")
rownames(aboInd) <- c("Stopped", "Continued")

# Perform Fisher's Exact Test
fisher_test <- fisher.test(aboInd)

# Output the results
fisher_test #p=0.08

table(combined_table$Abort, combined_table$Trial)



#-#-#-#-#-#-#-#-#-#-#-#-#-#-#-#-#-#-#-#-#-#-#-#-#-#-#-#-#-#-#-#-#-#-#-#-#
# 4. Models - Absolute (mean) difference Individual preference        ----
#-#-#-#-#-#-#-#-#-#-#-#-#-#-#-#-#-#-#-#-#-#-#-#-#-#-#-#-#-#-#-#-#-#-#-#-#

# Data file (as csv): "Capitain2025_FVets_ScentPreference_InputData_IndividualVariation.csv"
IndData <- data.frame(read.csv(file.choose(), sep = ",", dec = ".", header = T, stringsAsFactors=T))

#* Choice ----
wilcox.test(Abs_Tot_Choice_Diff~Group, data = IndData,
       exact = TRUE, correct = TRUE, conf.int = FALSE)
```

```
#* Commands ----
wilcox.test(Abs_Mean_Number.of.commands_Diff~Group, data = IndData,
        exact = TRUE, correct = TRUE, conf.int = FALSE)

#* Proximity ----
wilcox.test(Abs_Mean_Prox.target.GT.Prop_Test~Group, data = IndData,
        exact = TRUE, correct = TRUE, conf.int = FALSE)

#* Engaging ----
wilcox.test(Abs_Mean_Engaging.with.target.PropP_Test~Group, data = IndData,
        exact = TRUE, correct = TRUE, conf.int = FALSE)

#* Looking Guided ----
wilcox.test(Abs_Mean_Looking.at.target.GT.Prop_Guided~Group, data = IndData,
        exact = TRUE, correct = TRUE, conf.int = FALSE)

#* Looking Test ----
wilcox.test(Abs_Mean_Looking.at.target.GT.Prop_Test~Group, data = IndData,
        exact = TRUE, correct = TRUE, conf.int = FALSE)

#* Sniffing ----
wilcox.test(Abs_Mean_Sniffing.target.PropP_Guided~Group, data = IndData,
        exact = TRUE, correct = TRUE, conf.int = FALSE)

#* Latency ----
wilcox.test(Abs_Mean_LatencySmell_Test~Group, data = IndData,
        exact = TRUE, correct = TRUE, conf.int = FALSE)



#-#-###-#-#-#-#-#-#-#-#-#-#-#-#-#-#-##-#-#-#-#-#-#-#-#
# 5. PLOTS                              ----
#-#-###-#-#-#-#-#-#-#-#-#-#-#-#-#-#-##-#-#-#-#-#-#-#-#

# Reshape the Behaviour columns (Behaviour1 to BehaviourN) to long format
plotsum_data <- combined_table %>%
  select(AnimalID, Group, TrialType, Trial, Smell, Side.Behaviour,
       17:45) %>%
  gather(key = "Behaviour", value = "EventOccurred", -AnimalID, -Group, -TrialType, -Trial, -Smell, -
Side.Behaviour
  )

# convert structure
str(plotsum_data)
plotsum_data[, 1:6] <- lapply(plotsum_data[, 1:6], as.factor)
plotsum_data$EventOccurred <- as.numeric(plotsum_data$EventOccurred)
plotsum_data$EventOccurred[is.nan(plotsum_data$EventOccurred)] <- NA

# Summarize event occurrences by AnimalID, Group, Smell, and Behaviour
plot_summary_data <- plotsum_data %>%
  group_by(AnimalID, Group, TrialType, Trial, Smell, Behaviour, Side.Behaviour
  ) %>%
```

```
  summarise(
    EventCount = sum(EventOccurred, na.rm = TRUE))

plot_mean_summary_data <- plotsum_data %>%
  group_by(AnimalID, Group, TrialType, Smell, Behaviour, Side.Behaviour
  ) %>%
  summarise(
    EventCount = mean(EventOccurred, na.rm = TRUE))

plot_sum_summary_data <- plotsum_data %>%
  group_by(AnimalID, Group, TrialType, Smell, Behaviour, Side.Behaviour
  ) %>%
  summarise(
    EventCount = sum(EventOccurred, na.rm = TRUE))

# Prox experimenter.Prop
filtered_data_proxE <- plot_mean_summary_data %>%  filter(Behaviour ==
"Proximity.experimenter.Prop")
# Plot the event occurrences by Behaviour, Group, and Smell
ggplot(filtered_data_proxE, aes(x = Behaviour, y = EventCount, fill=Group)) +
  geom_boxplot(position = position_dodge(width = 2), size = 0.8) +
  #facet_wrap(~Group)+
  geom_jitter(aes(color = AnimalID), width = 0.1, alpha = 0.8, col="black") +
  labs(x="Group", y = "Mean proportion of time spent \n within 1m of the experimenter") +
  theme_minimal()+
  theme(
    axis.text.x = element_blank(),  # Remove x-axis labels (Behaviour)
    axis.ticks.x = element_blank(),  # Remove x-axis ticks
    strip.text = element_text(size = 14),  # Increase facet labels size
    axis.text = element_text(size = 14),  # Increase axis tick labels size
    axis.title = element_text(size = 16),  # Increase axis titles size
    plot.title = element_text(size = 18, face = "bold"),  # Increase plot title size
    panel.grid = element_blank(),  # Optional: Remove grid lines for cleaner look
    strip.position = "bottom",  # Move the facet labels to the bottom of the plot
    axis.title.y = element_text(margin = margin(r = 15))  # Increase right margin
  ) +
  facet_wrap(~Group, strip.position = "bottom") +  # Ensure facet labels are at the bottom
  scale_fill_manual(values = c("Control" = "lightgrey", "Experimental" = "darkgrey"))+   # Custom
colors
  ylim(0,0.8)


# Tail wag low binary
filtered_data <- plot_mean_summary_data %>%  filter(Behaviour == "Tail.wagging.low.Prop",
TrialType=="Test")
filtered_data <- filtered_data %>%
  mutate(EventCount = ifelse(EventCount != 0, 1, 0))
# Summarize the data: calculate the mean, SD, and SE of occurrences (x/8 for each individual)
summarized_data <- filtered_data %>%
  group_by(Group, Behaviour) %>%
  summarize(
    MeanOccurrence = mean(EventCount, na.rm = TRUE),
    SD = sd(EventCount, na.rm = TRUE),
```

```
    n = n(),  # Number of observations
    SE = SD / sqrt(n)  # Calculate the standard error
  )
# Now, create the ggplot with error bars for SE and custom colors
ggplot(summarized_data, aes(x = Behaviour, y = MeanOccurrence, fill = Group)) +
  geom_bar(stat = "identity", position = "dodge") +  # Bar plot for the mean
  geom_errorbar(
    aes(ymin = MeanOccurrence - SE, ymax = MeanOccurrence + SE),
    width = 0.2, size=1,   # Adjust the width of the error bars
    position = position_dodge(0.8)  # Align the error bars with the bars
  ) +
  facet_wrap(~Group) +
  scale_fill_manual(values = c("Control" = "lightgrey", "Experimental" = "darkgrey")) +  # Custom
colors
  labs(
    x = NULL,
    y = "Mean likelihood of low tail position"
  ) +
  theme_minimal() +
  theme(
    axis.text.x = element_blank(),  # Remove x-axis labels (Behaviour)
    axis.ticks.x = element_blank(),  # Remove x-axis ticks
    strip.text = element_text(size = 14),  # Increase facet labels size
    axis.text = element_text(size = 14),  # Increase axis tick labels size
    axis.title = element_text(size = 16),  # Increase axis titles size
    plot.title = element_text(size = 18, face = "bold"),  # Increase plot title size
    panel.grid = element_blank(),  # Optional: Remove grid lines for cleaner look
    axis.title.y = element_text(margin = margin(r = 15)),  # Increase right margin
    strip.position = "bottom"  # Move the facet labels to the bottom of the plot
  ) +
  facet_wrap(~Group, strip.position = "bottom")  # Ensure facet labels are at the bottom


# Sniffing target ~ Age
filtered_data_sniffTP <- plot_mean_summary_data %>%  filter(Behaviour == "Sniffing.target.PropP")
# Plot the event occurrences by Behaviour, Group, and Smell
ggplot(filtered_data_proxE, aes(x = Behaviour, y = EventCount, fill=Group)) +
  geom_boxplot(position = position_dodge(width = 2), size = 0.8) +
  #facet_wrap(~Group)+
  geom_jitter(aes(color = AnimalID), width = 0.1, alpha = 0.8, col="black") +
  labs(x="Group", y = "Mean proportion of time spent \n within 1m of the experimenter") +
  theme_minimal()+
  theme(
    axis.text.x = element_blank(),  # Remove x-axis labels (Behaviour)
    axis.ticks.x = element_blank(),  # Remove x-axis ticks
    strip.text = element_text(size = 14),  # Increase facet labels size
    axis.text = element_text(size = 14),  # Increase axis tick labels size
    axis.title = element_text(size = 16),  # Increase axis titles size
    plot.title = element_text(size = 18, face = "bold"),  # Increase plot title size
    panel.grid = element_blank(),  # Optional: Remove grid lines for cleaner look
    strip.position = "bottom",  # Move the facet labels to the bottom of the plot
    axis.title.y = element_text(margin = margin(r = 15))  # Increase right margin
  ) +
```

```
  facet_wrap(~Group, strip.position = "bottom") +  # Ensure facet labels are at the bottom
  scale_fill_manual(values = c("Control" = "lightgrey", "Experimental" = "darkgrey"))+   # Custom
colors
  ylim(0,0.8)


# Latency mean
combined_table %>%
  group_by(AnimalID, Smell, Group, TrialType) %>%
  summarise(mean.LatencySmell = mean(LatencySmell, na.rm = TRUE),
          median.LatencySmell = median(LatencySmell, na.rm=T),
          n_obs=length(na.omit(LatencySmell)),
          sd=sd(LatencySmell, na.rm=T),
          min.lat=min(LatencySmell, na.rm=T),
          max.lat=max(LatencySmell, na.rm=T)) %>%
  # mutate(group.smell = paste(Group, Smell, sep="_")) %>%
  filter(TrialType != "Guided",
       Smell != "none") %>% droplevels() %>%
  ggplot(aes(x = interaction(Smell, Group), y = mean.LatencySmell, fill=interaction(Smell,Group))) +
  geom_boxplot(aes(fill = interaction(Smell,Group)), alpha = 0.5, size=1) +
  geom_line(aes(group = interaction(AnimalID, Group)), colour = "darkgrey") +
  geom_point(aes(group = interaction(AnimalID, Group), color=AnimalID),
           alpha = 0.5, size=3) +
  theme_minimal() +
  ylim(0,23)+
  theme(
    axis.text.x = element_blank(),  # Remove x-axis labels (Behaviour)
    axis.ticks.x = element_blank(),  # Remove x-axis ticks
    strip.text = element_text(size = 14),  # Increase facet labels size
    axis.text = element_text(size = 14),  # Increase axis tick labels size
    axis.title = element_text(size = 16),  # Increase axis titles size
    plot.title = element_text(size = 18, face = "bold"),  # Increase plot title size
    panel.grid = element_blank(),  # Optional: Remove grid lines for cleaner look
    axis.title.y = element_text(margin = margin(r = 15)),  # Increase right margin
    strip.position = "bottom"  # Move the facet labels to the bottom of the plot
  ) +
  labs(
    x = "     Control         Experimental",
    y = "Mean latency to \n accomplish command (s)      "
  ) +
  scale_fill_manual(values = c(      # Custom colors
    "Smell1.Control" = "white",
    "Smell1.Experimental" = "white",
    "Smell2.Control" = "grey45",
    "Smell2.Experimental" = "brown"
  ))


# Commands

# Create the 'command.smell' column, where command number value is placed in rows where
Command.accomplished == 1
combined_table <- combined_table %>%
```

```
  group_by(TrialNo, AnimalID) %>%
  mutate(
    # For rows where Command.accomplished == 1, get the Latency.to.accomplish.command value
from the same TrialNo and AnimalID group
    Command.Smell = ifelse(
      Command.accomplished == 1,
      first(Number.of.commands[!is.na(Number.of.commands)]),
      NA )
  ) %>%
  ungroup()

combined_table %>%
  group_by(AnimalID, Smell, Group, TrialType) %>%
  summarise(sum.Command.Smell = sum(Command.Smell, na.rm = TRUE),
          mean.Command.Smell = mean(Command.Smell, na.rm=T),
          median.Command.Smell = median(Command.Smell, na.rm=T),
          n_obs=length(na.omit(Command.Smell)),
          sd=sd(Command.Smell, na.rm=T),
          min.lat=min(Command.Smell, na.rm=T),
          max.lat=max(Command.Smell, na.rm=T)) %>%
  # mutate(group.smell = paste(Group, Smell, sep="_")) %>%
  filter(TrialType != "Guided",
       Smell != "none") %>% droplevels() %>%
  ggplot(aes(x = interaction(Smell, Group), y = mean.Command.Smell, fill=interaction(Smell,Group))) +
  geom_boxplot(aes(fill = interaction(Smell,Group)), alpha = 0.5, size=1) +
  geom_line(aes(group = interaction(AnimalID, Group)), colour = "darkgrey") +
  geom_point(aes(group = interaction(AnimalID, Group), color=AnimalID),
           alpha = 0.5, size=3) +
  theme_minimal() +
  theme(
    axis.text.x = element_blank(),  # Remove x-axis labels (Behaviour)
    axis.ticks.x = element_blank(),  # Remove x-axis ticks
    strip.text = element_text(size = 14),  # Increase facet labels size
    axis.text = element_text(size = 14),  # Increase axis tick labels size
    axis.title = element_text(size = 16),  # Increase axis titles size
    plot.title = element_text(size = 18, face = "bold"),  # Increase plot title size
    panel.grid = element_blank(),  # Optional: Remove grid lines for cleaner look
    axis.title.y = element_text(margin = margin(r = 15)),  # Increase right margin
    strip.position = "bottom"  # Move the facet labels to the bottom of the plot
  ) +
  ylim(0, 7)+
  scale_y_continuous(breaks = seq(0,7, by = 1)) +
  labs(
    x = "Control                                Test",
    y = "Mean number of commands      "
  ) +
  scale_fill_manual(values = c(      # Custom colors
    "Smell1.Control" = "white",
    "Smell1.Experimental" = "white",
    "Smell2.Control" = "grey45",
    "Smell2.Experimental" = "brown"
  ))
```

```
#-#-#-#-#-#-#-#-#-#-#-#-#-#-#-#-#-#-#-#-#-#-#
#     Predefine functions              #####
#-#-#-#-#-#-#-#-#-#-#-#-#-#-#-#-#-#-#-#-#-#-#

overdisp.test <- function(model) {
  # Extract Pearson residuals
  res <- residuals(model, type = "pearson")
  rdf <- df.residual(model)
  rp <- sum(res^2) / rdf
  p <- pchisq(rp * rdf, rdf, lower.tail = FALSE)
  return(list(dispersion = rp, p_value = p))
}

#transform the response because beta cannot handle 0 and 1
beta.tr<-function(x){
  if(any(is.na(x))){warning("x comprises NAs")}
  return((x*(length(x) - 1) + 0.5)/length(x))
}
```
