# Supplementary material for: Not just avoidance: dogs show subtle individual differences in reacting to human fear chemosignals
Source: Front Vet Sci. 2025 Sep 15;12:1679991. doi: 10.3389/fvets.2025.1679991 (PMC12477697; doi:10.3389/fvets.2025.1679991)
Supplement: Supplementary file 5 [file Data_Sheet_2.PDF]

Supplementary Material

Title: *Not just avoidance: Dogs show subtle individual differences in reacting to human fear chemosignals*

Authors: Svenja Capitain[1*], Friederike Range[1], & Sarah Marshall-Pescini[1]

[1] Domestication Lab, Konrad Lorenz Institute of Ethology, University of Veterinary Medicine Vienna, Austria

* svenja.capitain@vetmeduni.ac.at, friederike.range@vetmeduni.ac.at

Published in: Frontiers in Veterinary Science, 2025

Content

1 Associated supplementary files 2
2 Sample collection – movie scenes 3
3 Analysis of anxiety scores 3
4 Sample processing 3
5 Interrater reliability 4
6 Detailed model outputs 5
6.1 Target-directed behaviours: Smell*Group 5
6.1.1 CHOICE 5
6.1.2 FIRST CHOICE 6
6.1.3 SIDE BIAS 7
6.1.4 PROXIMITY TO TARGET 7
6.1.5 SNIFFING TARGET 8
6.1.6 ENGAGING WITH TARGET 9
6.1.7 LOOKING AT TARGET 10
6.1.8 NUMBER OF COMMANDS 10
6.1.9 LATENCY TO ACCOPMLISH COMMAND 11
6.2 Target-directed behaviors – Experimental group: Smell*(Age+Sex) 13
6.2.1 CHOICE 13
6.2.2 FIRST CHOICE 14
6.2.3 PROXIMITY TO TARGET 15
6.2.4 SNIFFING TARGET 16
6.2.5 ENGAGING WITH TARGET 17
6.2.6 LOOKING AT TARGET 18
6.2.7 NUMBER OF COMMANDS 19

6.2.8 LATENCY TO ACCOMPLISH COMMANDS 19
6.3 Non-target directed behaviors 20
6.3.1 SNIFFING ENVIRONMENT 20
6.3.2 LOOKING AT EXPERIMENTER 21
6.3.3 PROXIMITY TO EXPERIMENTER 22
6.3.4 TAIL HIGH 23
6.3.5 TAIL LOW 24
6.3.6 TRIAL NOT ACCOMPLISHED 25
6.3.7 SESSION TERMINATED 26

1 Associated supplementary files

Data (including data description)

- File “Capitain2025_Fvets_ ScentPreference_InputData_GroupComparison.xlsx”
    - Dog demographics (breed, age, sex)
    - Raw data and proportions for each behavior, trial, and smell
- File “Capitain2025_Fvets_ ScentPreference_InputData_IndividualVariation.xlsx”
    - Prepared data for the comparison of individual differences between smells

Statistical Analysis

- File “Capitain2025_ Fvets_ScentPreference_AnalysScript_Rev1.docx”
    - Statistical analysis script of the group analysis and individual variation analysis

Video S1

- File “Capitain2025_ Fvets_ScentPref_VideoS1.mp4”
    - Visual presentation of the trial procedure and different dogs’ reaction

Table B

- File “Capitain2025_FVets_ScentPreference_SupplementTableB_DogChoiceOverview.xlsx”
    - Overview of the number and percentage of choices each dog made per side and smell

2 Sample collection – movie scenes

Neutral movie scenes were taken from and validated in de Groot, J. H., Smeets, M. A., & Semin, G. R. (2015). *Rapid stress system drives chemical transfer of fear from sender to receiver*. PLOS ONE, 10(2), e0118211. https://doi.org/10.1371/journal.pone.0118211.

- We selected two clips for the neutral condition: Clip 23t (8 min): a first-person view of a car and boat traveling through the Dutch countryside. Clip 27t (13 min, 26 s): an excerpt from the TV show "Rail Away", displayed a train traveling through the Alps.

Fear movie scenes were validated in de Groot, J. H., Kirk, P. A., & Gottfried, J. A. (2020). *Encoding fear intensity in human sweat*. Philosophical Transactions of the Royal Society B, 375(1800), 20190271. https://doi.org/10.1098/rstb.2019.0271. They were made available online by de Groot, J. H., Kirk, P. A., & Gottfried, J. A. (2021). *Titrating the smell of fear: Initial evidence for dose-invariant behavioral, physiological, and neural responses*. Psychological Science, 32(4), 558-572. https://doi.org/10.1177/0956797620970548.

- We selected four clips for the fear condition: Clip f12 (3 min, 22 s): "The passenger". Clip f8 (4 min, 35 s): "Nighty Night, Nancy". Clip f4 (11 min, 25 s): "Vicious". Clip f9 (3 min, 28 s): "Mr Creak".

3 Analysis of anxiety scores

Odour donators filled out the Spielberger's State-Trait Anxiety Inventory (Spielberg et al., 1971, German translation from Grimm, 2009), a 10-item questionnaire assessing the current relaxation and nervousness-level across 7-point Likert scales, right before and after watching each movie. A paired t-test was used for each participant and condition to assess whether the scores before and after watching a movie were significantly different. All participants were significantly more stressed after watching the fear movie ($p<0.02$), while this was not the case for anyone after watching the neutral movie ($p>0.05$).

Spielberger CD, Gonzalez-Reigosa F, Martinez-Urrutia A, Natalicio LF, Natalicio DS. The state-trait anxiety inventory. Revista Interamericana de Psicologia/Interamerican journal of psychology. 1971;5(3 & 4).

Grimm J. State-Trait-Anxiety Inventory according to Spielberger. German long and short version.-Methods Forum of the University of Vienna. MF-Working Paper 2009/02; 2009.

4 Sample processing

Each odour donor donated eight samples (one pad per axillary, each cut into four pieces) per movie – two times neutral odour or once neutral and once fear. Each piece was placed in a sample tube which was labelled with the condition (Neutral 1/ Neutral2/Fear). At the freezer, the sample was moved into a different sampling tube that held a piece from a different person in the same condition. The tube was labelled with a number and a star or a 0. The number and donator identity were not connected on the sampling sheet, allowing for full anonymity. A sample from the same two people but the other sampling (first vs second neutral movie or neutral vs fear movie) was placed in a second tube with the same number and a 0 or star respectively. Which numbers were combined and which number and star/0 combination connected to which smell and condition was noted in a master sheet. Which smell (Neutral 1/ Neutral2/Fear) was inside the tube was noted on the tube as well, but covered with a sticker. The smell collection and processing commenced five weeks before the start of the experiment.

On the testing sheet, the tube numbers – without the information of their content or condition – were connected to the trial sequence: in what sequence the targets were exchanged between the left and right side during the ten test trials, and whether the dog was led to the left or right first during the guided phase. Upon recruitment, dogs were randomly allocated to a sample pair number and thereby, blindly, to a group. A second person, unaware of the hypotheses and not part of the testing, checked after every five dogs if age and breed were somewhat balanced across groups and switched dogs to a different number if that was not the case.

On the test day, the experimenter took the sample tube pair with the number indicated in the testing sheet out of the freezer for the respective dog. The tube with the star was always placed in the left target first, and the trial sequence indicated in the sheet was followed. The experimenter was hence completely blind which group the dog belonged to and which target held which smell. After the test, to counteract problems due to possible mistakes during the target switching, the owners took off the stickers that covered the condition and they were held into the camera with the info which side each tube was in in the last trial. This info was saved separately from the test video for the coding. This way, the experimenter continued to be blind to smells and conditions during coding. But, after the coding, it could be verified that the smells had ended up in the correct sequence as they had been indicated on the sampling sheet. This procedure revealed that no mistakes had been made.

5 Interrater reliability

Supplement Table A: Inter-class correlation coefficients (ICC) for each behavior. Twenty-one percent of all videos (n=13 videos) were re-coded by a second, hypothesis-blind coder who was likewise blind to condition, group, and smell location.

**Guided phase**	**ICC**	**Test phase**	**ICC**
Looking at target (D)	0.89	Looking at target (D)	0.95
Sniffing target (D)	0.95	Engaging with target (D)	0.92
Proximity to target (D)	0.82	Proximity to target (D)	0.83
		Latency to accomplish command (D)	0.97
		Command accomplished (F/B)	0.98
		Not accomplished (F/B)	1.00
		Redirection (F)	1.00
		Number of commands (F)	1.00
		Tail high (D)	0.95
		Tail low (D)	0.92
		Looking at experimenter (D)	0.96
		Proximity to experimenter (D)	0.99
		Sniffing environment (D)	0.96
		Trial duration	0.98
**Total**			**0.95**

6 Detailed model outputs

Detailed model outputs for each model referenced in the result section. Includes model formula, full-null model comparison (using a likelihood ratio test, with the null model retaining control predictors and random effects), model summary, Tukey-adjusted pairwise comparisons (emmeans) where applicable, and 95% Confidence Intervals (Parametric bootstrapping).

6.1 Target-directed behaviours: Smell*Group

6.1.1 CHOICE

Formula: Command.accomplished ~ Smell * Group + Age.z + Sex + Trial.z + offset(Total_Choice_log) + (1 + Smell2 + Trial.z || AnimalID), family = binomial

Conditional model – Summary

term	estimate	std.error	statistic	p.value
(Intercept)	-2.195	0.169	-13.007	0.000
SmellSmell2	0.230	0.240	0.958	0.338
GroupExperimental	0.127	0.195	0.651	0.515
Trial.z	-0.128	0.060	-2.118	0.034
Age.z	-0.020	0.060	-0.327	0.744
SexM	-0.008	0.124	-0.066	0.948
SmellSmell2:GroupExperimental	-0.183	0.296	-0.619	0.536

	npar	AIC	BIC	logLik	deviance	Chisq	Df	Pr(>Chisq)
null.Choice	7	1588.69	1624.07	-787.34	1574.69			
full.Choice	13	1596.40	1662.11	-785.20	1570.40	4.29	6.00	0.64

Confidence intervals

	orig	LOWER CI (2.5%)	UPPER CI (97.5%)
(Intercept)	-2.195	-2.458	-1.891
SmellSmell2	0.230	-0.230	0.727
GroupExperimental	0.127	-0.190	0.445
Age.z	-0.128	-0.240	-0.014
SexM	-0.020	-0.120	0.100

Trial.z	-0.008	-0.191	0.235
SmellSmell2:GroupExperimental	-0.183	-0.718	0.307

6.1.2 FIRST CHOICE

Formula: Command1.accomplished ~ Smell * Group + Age.z + Sex + Trial.z + (1 + Smell2 + Trial.z || AnimalID), family = binomial

Conditional model – Summary

term	estimate	std.error	statistic	p.value
(Intercept)	-0.619	0.506	-1.223	0.221
SmellSmell2	1.238	0.666	1.859	0.063
GroupExperimental	0.374	0.567	0.659	0.510
Trial.z	0.000	0.187	0.000	1.000
Age.z	0.000	0.382	0.000	1.000
SexM	-0.748	0.799	-0.936	0.350
SmellSmell2:GroupExperimental	-0.619	0.506	-1.223	0.221

	npar	AIC	BIC	logLik	deviance	Chisq	Df	Pr(>Chisq)
null.Choice	5	179.128	193.148	-84.564	169.128			
full.Choice	8	180.249	202.681	-82.125	164.249	4.879	3.000	0.181

Confidence intervals

	orig	LOWER CI (2.5%)	UPPER CI (97.5%)
(Intercept)	-0.619	-1.857	0.561
SmellSmell2	1.238	-0.212	3.228
GroupExperimental	0.374	-0.809	1.497
Age.z	0.000	-0.378	0.385
SexM	0.000	-0.711	0.779
Trial.z	-0.748	-2.730	1.156
SmellSmell2:GroupExperimental	-0.619	-1.857	0.561

6.1.3 SIDE BIAS

Fisher's Exact Test for Count Data

data: sidebiasInd

p-value = 0.5838

alternative hypothesis: true odds ratio is not equal to 1

95 percent confidence interval: 0.2090687 2.4242512

sample estimates: odds ratio 0.7092636

6.1.4 PROXIMITY TO TARGET

Formula: Proximity.target.GT.Prop ~ Smell * Group + Age.z + Sex + Trial.z +

(1 + Smell.Smell2 + Trial.z || AnimalID), family = beta

Conditional model – Summary

	Estimate	Std. Error	z-value
(Intercept)	0.831	0.071	11.687
SmellSmell2	-0.044	0.046	-0.942
GroupExperimental	0.042	0.078	0.544
Age.z	0.024	0.033	0.719
SexM	-0.016	0.066	-0.238
Trial.z	-0.028	0.017	-1.645
SmellSmell2:GroupExperimental	0.032	0.057	0.564

Null model comparison

	Df	AIC	BIC	logLik	deviance	Chisq	Df	Pr(>Chisq)
null.proximity	11	342.618	389.987	-160.309	320.618			
full.proximityP	14	346.757	407.045	-159.378	318.757	1.861	3.000	0.602

Confidence intervals

	LOWER CI (2.5%)	UPPER CI (97.5%)
cond@(Intercept)	0.681	0.977
cond@SmellSmell2	-0.131	0.048
cond@GroupExperimental	-0.114	0.197

cond@Age.z	-0.047	0.092
cond@SexM	-0.158	0.118
cond@Trial.z	-0.060	0.003
cond@SmellSmell2:GroupExperimental	-0.082	0.145
disp@(Intercept)	0.681	0.977

6.1.5 SNIFFING TARGET

Formula: Sniffing.target.PropP ~ Smell * Group + Age.z + Sex + Trial.z +
(1 + Smell.Smell2 + Trial.z || AnimalID), family = beta

Conditional model – Summary

	Estimate	Std. Error	z-value	Pr(>|z|)	
(Intercept)	-1.065	0.180	-5.906	0.000	***
SmellSmell2	0.031	0.114	0.275	0.783	
GroupExperimental	-0.149	0.194	-0.768	0.443	
Age.z	-0.209	0.085	-2.469	0.014	*
SexM	0.012	0.174	0.067	0.946	
Trial.z	-0.313	0.056	-5.550	0.000	***
SmellSmell2:GroupExperimental	0.055	0.141	0.393	0.695	

Null model comparison

	Df	AIC	BIC	logLik	deviance	Chisq	Df	Pr(>Chisq)
null.SniffTP	8	-2428.7	-2388.54	1222.37	-2444.74			
full.sniffingTP	11	-2424.4	-2369.09	1223.18	-2446.37	1.631	3.000	0.652

Confidence intervals

	LOWER CI (2.5%)	UPPER CI (97.5%)
cond@(Intercept)	-1.419	-0.689
cond@SmellSmell2	-0.193	0.256
cond@GroupExperimental	-0.548	0.223
cond@Age.z	-0.373	-0.047
cond@SexM	-0.324	0.351

cond@Trial.z	-0.421	-0.208
cond@SmellSmell2:GroupExperimental	-0.225	0.341
disp@(Intercept)	0.500	0.673

6.1.6 ENGAGING WITH TARGET

Formula: Engaging.with.target.PropP ~ Smell * Group + Age.z + Sex + Trial.z +
(1 + Smell.Smell2 + Trial.z || AnimalID), family = beta

Conditional model – Summary

	Estimate	Std. Error	z-value	Pr(>|z|)
(Intercept)	-0.285	0.242	-1.178	0.239
SmellSmell2	0.011	0.211	0.054	0.957
GroupExperimental	-0.124	0.250	-0.495	0.620
Age.z	0.120	0.102	1.174	0.240
SexM	0.275	0.208	1.327	0.185
Trial.z	-0.040	0.071	-0.564	0.572
SmellSmell2:GroupExperimental	0.011	0.251	0.045	0.964

Null model comparison

	Df	AIC	BIC	logLik	deviance	Chisq	Df	Pr(>Chisq)
null.EngagTP	8	-71.946	-43.308	43.973	-87.946			
full.EngagingTP	11	-66.258	-26.881	44.129	-88.258	0.312	3.000	0.958

Confidence intervals

	LOWER CI (2.5%)	UPPER CI (97.5%)
cond@(Intercept)	-0.790	0.204
cond@SmellSmell2	-0.397	0.450
cond@GroupExperimental	-0.627	0.381
cond@Age.z	-0.074	0.321
cond@SexM	-0.121	0.682
cond@Trial.z	-0.183	0.098
cond@SmellSmell2:GroupExperimental	-0.499	0.496
disp@(Intercept)	1.465	1.876

6.1.7 LOOKING AT TARGET

Formula: Looking.at.target.PropP ~ Smell * Group + Age.z + Sex + Trial.z +
(1 + Smell.Smell2 + Trial.z || AnimalID), family = beta

Conditional model – Summary

	Estimate	Std. Error	z-value	Pr(>|z|)	
(Intercept)	-1.392	0.083	-16.691	0.000	***
SmellSmell2	0.005	0.060	0.083	0.934	
GroupExperimental	0.030	0.090	0.334	0.738	
Age.z	-0.041	0.039	-1.066	0.287	
SexM	0.012	0.079	0.151	0.880	
Trial.z	-0.096	0.019	-5.039	0.000	***
SmellSmell2:GroupExperimental	0.035	0.074	0.480	0.631	

Null model comparison

	Df	AIC	BIC	logLik	deviance	Chisq	Df	Pr(>Chisq)
null.LookTP	-2521.4	-2477.7	1268.700	-2537.4				-2521.401
full.LookingTP	-2516.6	-2456.5	1269.308	-2538.6	1.215	3.000	0.749	-2516.615

Confidence intervals

	LOWER CI (2.5%)	UPPER CI (97.5%)
cond@(Intercept)	-1.550	-1.230
cond@SmellSmell2	-0.117	0.116
cond@GroupExperimental	-0.139	0.201
cond@Age.z	-0.119	0.034
cond@SexM	-0.124	0.154
cond@Trial.z	-0.134	-0.059
cond@SmellSmell2:GroupExperimental	-0.100	0.187
disp@(Intercept)	2.154	2.294

6.1.8 NUMBER OF COMMANDS

Formula: Commands.Smell ~ Smell * Group + Age.z + Sex + Trial.z +

(1 + Smell2 + Trial.z || AnimalID), family = poisson

Conditional model – Summary

	Estimate	Std. Error	z-value	Pr(>|z|)	
(Intercept)	0.448	0.113	3.970	0.000	***
SmellSmell2	0.121	0.121	1.003	0.316	
GroupExperimental	0.177	0.123	1.439	0.150	
Age.z	0.038	0.048	0.796	0.426	
SexM	0.015	0.096	0.157	0.875	
Trial.z	0.083	0.032	2.579	0.010	*
SmellSmell2:GroupExperimental	-0.141	0.147	-0.960	0.337	

Null model comparison

	npar	AIC	BIC	logLik	deviance	Chisq	Df	P-value
null.Commands	7	1686.8	1716.79	-836.3	1672.79			
full.Commands	10	1690.6	1733.45	-835.2	1670.59	2.201	3.000	0.532

Confidence intervals

	LOWER CI (2.5%)	UPPER CI (97.5%)
(Intercept)	0.192	0.664
SmellSmell2	-0.120	0.332
GroupExperimental	-0.042	0.408
Age.z	-0.042	0.130
SexM	-0.172	0.290
Trial.z	0.024	0.153
SmellSmell2:GroupExperimental	-0.441	0.138

6.1.9 LATENCY TO ACCOPMLISH COMMAND

Formula: LatencySmell_log ~ Smell * Group + Age.z + Sex + Trial.z +

(1 + Smell.Smell2 + Trial.z || AnimalID), family = gaussian

Conditional model – Summary

	Estimate	Std. Error	t-value

(Intercept)	2.507	0.236	10.601
GroupExperimental	0.278	0.267	1.04
SmellSmell2	0.083	0.091	0.912
Age.z	0.016	0.052	0.313
SexM	-0.080	0.107	-0.75
Trial.z	0.067	0.030	2.221
GroupExperimental:SmellSmell1	-0.085	0.262	-0.324
GroupExperimental:SmellSmell2	-0.098	0.267	-0.368

Drop1

	npar	AIC	LRT	Pr(Chi)	
<none>	1069.8				
Age.z	1	1067.9	0.102	0.749	
Sex	1	1068.4	0.560	0.454	
Trial.z	1	1072.5	4.639	0.031	*
Group:Smell	2	1066	0.131	0.936	

Null model comparison

	npar	AIC	BIC	logLik	deviance	Chisq	Df	Pr(>Chisq)	
null.lat	8	1135.4	1170.1	-559.7	1119.4				
full.lat	16	1069.8	1139.2	-518.91	1037.8	81.563	8	2.37E-14	***

Reduced model

Formula: LatencySmell_log ~ Smell + Group + Age.z + Sex + Trial.z +

(1 + Smell.Smell2 + Trial.z || AnimalID), family = gaussian

Conditional model – Summary

	Estimate	Std. Error	t-value
(Intercept)	2.611	0.150	17.457
GroupExperimental	0.188	0.109	1.727
SmellSmell2	0.067	0.053	1.277
Age.z	0.029	0.051	0.571

SexM	-0.119	0.104	-1.141
Trial.z	0.064	0.029	2.200

Confidence intervals

	LOWER CI (2.5%)	UPPER CI (97.5%)
(Intercept)	2.332	2.902
GroupExperimental	-0.019	0.379
SmellSmell2	-0.021	0.081
Age.z	-0.070	0.127
SexM	-0.326	0.080
Trial.z	0.007	0.124

Posthoc

Group	emmean	SE	df	lower.CL	upper.CL	p-value
Control	1.836	0.094	69.824	1.648	2.024	
Test	2.023	0.069	76.696	1.885	2.161	0.08

6.2 Target-directed behaviors – Experimental group: Smell*(Age+Sex)

6.2.1 CHOICE

Formula: Command.accomplished ~ Smell* (Age.z + Sex) + Trial.z + offset(TotalChoices_log) + (1+Smell.Smell2+Trial.z|AnimalID), family=binomial

Conditional model – Summary

	Estimate	Std. Error	t-value	P-value	
(Intercept)	-2.03	0.14	-14.23	<2e-16	***
SmellSmell2	0.00	0.22	0.00	1.00	
Age.z	0.01	0.11	0.09	0.93	
SexM	-0.10	0.24	-0.40	0.69	
Trial.z	-0.16	0.07	-2.17	0.03	*
SmellSmell2:Age.z	-0.06	0.17	-0.35	0.73	
SmellSmell2:SexM	0.12	0.36	0.34	0.74	

Null model comparison

	npar	AIC	BIC	logLik	deviance	Chisq	Df	Pr(>Chisq)
null.c	5	1045.7	1068.9	-517.9	1035.7			
full.c	13	1059.2	1119.5	-516.6	1033.2	2.5	8	0.962

	LOWER CI (2.5%)	UPPER CI (97.5%)
(Intercept)	-2.30	-1.78
SmellSmell2	-0.37	0.46
Age.z	-0.28	0.22
SexM	-0.54	0.41
Trial.z	-0.33	-0.03
SmellSmell2:Age.z	-0.38	0.28
SmellSmell2:SexM	-0.76	0.85
(Intercept)	-2.30	-1.78

6.2.2 FIRST CHOICE

Formula: Command.accomplished ~ Smell*(Age.z + Sex) + (1+Smell.Smell2||AnimalID), family=binomial

Conditional model – Summary

	Estimate	Std. Error	t-value	P-value
(Intercept)	0.00	0.39	0.00	1.00
SmellSmell2	0.00	0.55	0.00	1.00
Age.z	-0.02	0.32	-0.07	0.95
SexM	-0.69	0.67	-1.03	0.30
SmellSmell2:Age.z	0.04	0.46	0.10	0.92
SmellSmell2:SexM	1.38	0.95	1.45	0.15

Null model comparison

	npar	AIC	BIC	logLik	deviance	Chisq	Df	Pr(>Chisq)
null.fc	3	119.68	126.9	-56.83	113.68			
full.fc	8	126.27	145.52	-55.13	110.27	3.4074	5	0.64

Confidence interval

	LOWER CI (2.5%)	UPPER CI (97.5%)
(Intercept)	-0.87	0.85
SmellSmell2	-1.22	1.36
Age.z	-0.91	0.60
SexM	-2.63	0.60
SmellSmell2:Age.z	-1.01	1.14
SmellSmell2:SexM	-0.35	4.12

6.2.3 PROXIMITY TO TARGET

Formula: Proximity.to.target_log ~ Smell * (Age.z + Sex) + Trial.z + (1+Smell.Smell2+Trial.z|AnimalID),

Conditional model – Summary

	Estimate	Std. Error	t-value
(Intercept)	0.86	0.06	13.96
SmellSmell2	0.03	0.05	0.70
Age.z	0.01	0.05	0.15
SexM	0.03	0.10	0.25
Trial.z	-0.04	0.03	-1.61
SmellSmell2:Age.z	0.00	0.03	-0.09
SmellSmell2:SexM	-0.10	0.07	-1.46

Null model comparison

	npar	AIC	BIC	logLik	deviance	Chisq	Df	Pr(>Chisq)
null.proximity.E	9	211.89	246.63	-96.943	193.89			
full.proximity.E	14	219.59	273.64	-95.795	191.59	2.2957	5	0.81

Confidence interval

	LOWER CI (2.5%)	UPPER CI (97.5%)
(Intercept)	0.75	1.01

SmellSmell2	-0.07	0.10
Age.z	-0.08	0.12
SexM	-0.19	0.21
Trial.z	-0.08	0.00
SmellSmell2:Age.z	-0.08	0.06
SmellSmell2:SexM	-0.23	0.05

6.2.4 SNIFFING TARGET

Formula: Sniffing.target.PropP ~ Smell * (Age.z + Sex) + Trial.z + (1+Smell.Smell2+Trial.z||AnimalID), family = beta_family

Conditional model – Summary

	Estimate	Std. Error	t-value	p-value	
(Intercept)	-1.23	0.13	-9.42	<0.01	***
SmellSmell2	0.10	0.10	0.96	0.34	
Age.z	-0.12	0.10	-1.18	0.24	
SexM	-0.04	0.21	-0.18	0.86	
Trial.z	-0.31	0.06	-5.12	<0.00	***
SmellSmell2:Age.z	0.02	0.08	0.20	0.84	
SmellSmell2:SexM	-0.02	0.17	-0.11	0.91	

Null model comparison

	npar	AIC	BIC	logLik	deviance	Chisq	Df	Pr(>Chisq)
null.SniffTP.E	6	-1421.7	-1394	716.87	-1433.7			
full.sniffingTP.E	11	-1414.5	-1363.7	718.25	-1436.5	2.7725	5	0.74

Confidence interval

	LOWER CI (2.5%)	UPPER CI (97.5%)
(Intercept)	-1.50	-0.97
SmellSmell2	-0.10	0.30
Age.z	-0.32	0.08
SexM	-0.46	0.39

Trial.z	-0.43	-0.19
SmellSmell2:Age.z	-0.14	0.17
SmellSmell2:SexM	-0.34	0.31

6.2.5 ENGAGING WITH TARGET

Formula: Engaging.with.target.PropP ~ Smell * (Age.z + Sex) + Trial.z + (1+Smell.Smell2+Trial.z||AnimalID), family = beta_family

Conditional model – Summary

	Estimate	Std. Error	t-value	p-value	
(Intercept)	-0.44	0.18	-2.48	0.01	*
SmellSmell2	-0.01	0.19	-0.05	0.96	
Age.z	0.18	0.13	1.31	0.19	
SexM	0.35	0.28	1.28	0.20	
Trial.z	-0.01	0.09	-0.09	0.93	
SmellSmell2:Age.z	-0.11	0.14	-0.74	0.46	
SmellSmell2:SexM	0.10	0.29	0.34	0.74	

Null model comparison

	npar	AIC	BIC	logLik	deviance	Chisq	Df	Pr(>Chisq)
null.SniffTP.E	6	-43.61	-24.29	27.80	-55.61			
full.sniffingTP.E	11	-38.27	-2.85	30.14	-60.27	4.66	5	0.46

Confidence interval

	LOWER CI (2.5%)	UPPER CI (97.5%)
(Intercept)	-0.78	-0.09
SmellSmell2	-0.37	0.36
Age.z	-0.09	0.44
SexM	-0.19	0.90
Trial.z	-0.18	0.16
SmellSmell2:Age.z	-0.38	0.17
SmellSmell2:SexM	-0.47	0.67

6.2.6 LOOKING AT TARGET

Formula: Looking.at.target.GT.Prop ~ Smell * (Age.z + Sex) + Trial.z + (1+Smell.Smell2+Trial.z||AnimalID), family = beta_family

Conditional model – Summary

	Estimate	Std. Error	t-value	p-value	
(Intercept)	-1.35	0.07	-19.58	<0.01	***
SmellSmell2	0.04	0.05	0.81	0.42	
Age.z	-0.07	0.05	-1.29	0.20	
SexM	-0.04	0.11	-0.34	0.73	
Trial.z	-0.11	0.02	-4.70	0.001	***
SmellSmell2:Age.z	0.02	0.04	0.40	0.69	
SmellSmell2:SexM	-0.01	0.09	-0.09	0.93	

Null model comparison

	npar	AIC	BIC	logLik	deviance	Chisq	Df	Pr(>Chisq)
null.SniffTP.E	6	-1624.5	-1594.4	818.27	-1636.5			
full.sniffingTP.E	11	-1617.3	-1562	819.64	-1639.3	2.723	5	0.74

Confidence interval

	LOWER CI (2.5%)	UPPER CI (97.5%)
(Intercept)	-1.48	-1.21
SmellSmell2	-0.06	0.15
Age.z	-0.18	0.04
SexM	-0.26	0.18
Trial.z	-0.15	-0.06
SmellSmell2:Age.z	-0.07	0.10
SmellSmell2:SexM	-0.18	0.17

6.2.7 NUMBER OF COMMANDS

Formula: Command.Smell ~ Smell * (Age.z + Sex) + Trial.z + (1+Smell.Smell2+Trial.z||AnimalID), family = poisson()

Conditional model – Summary

	Estimate	Std. Error	t-value	p-value	
(Intercept)	0.56	0.09	6.49	0.00	***
SmellSmell2	0.03	0.10	0.26	0.79	
Age.z	0.13	0.07	1.90	0.06	.
SexM	0.10	0.14	0.75	0.45	
Trial.z	0.10	0.04	2.49	0.01	*
SmellSmell2:Age.z	-0.10	0.08	-1.17	0.24	
SmellSmell2:SexM	-0.17	0.17	-1.01	0.31	

Null model comparison

	npar	AIC	BIC	logLik	deviance	Chisq	Df	Pr(>Chisq)
null.Commands.E	5	1071.8	1091.1	-530.89	1061.8			
full.Commands.E	10	1076.8	1115.5	-528.42	1056.8	4.9454	5	0.42

Confidence interval

	LOWER CI (2.5%)	UPPER CI (97.5%)
(Intercept)	0.39	0.73
SmellSmell2	-0.17	0.23
Age.z	0.00	0.26
SexM	-0.17	0.38
Trial.z	0.02	0.18
SmellSmell2:Age.z	-0.26	0.06
SmellSmell2:SexM	-0.49	0.16

6.2.8 LATENCY TO ACCOMPLISH COMMANDS

Formula: LatencySmell_log ~ Smell* (Age.z + Sex) + Trial.z + (1+Smell.Smell2+Trial.z||AnimalID)

Conditional model – Summary

	Estimate	Std. Error	t-value
(Intercept)	1.65	0.09	18.85
SmellSmell2	0.17	0.09	2.04
Age.z	0.07	0.07	1.00
SexM	-0.01	0.15	-0.06
Trial.z	0.09	0.04	2.22
SmellSmell2:Age.z	0.05	0.07	0.70
SmellSmell2:SexM	-0.30	0.14	-2.10

Null model comparison

	npar	AIC	BIC	logLik	deviance	Chisq	Df	Pr(>Chisq)
null.lat.E	8	685.26	716.17	-334.63	669.26			
full.lat.E	11	685.58	728.08	-331.79	663.58	5.6735	3	0.13

Confidence interval

	LOWER CI (2.5%)	UPPER CI (97.5%)
(Intercept)	2.32	2.90
GroupExperimental	-0.03	0.40
SmellSmell1	-1.33	-0.88
SmellSmell2	-1.27	-0.81
Age.z	-0.07	0.13
SexM	-0.32	0.09
Trial.z	0.01	0.12

6.3 Non-target directed behaviors

6.3.1 SNIFFING ENVIRONMENT

Formula: Sniffing.environment.Binary ~ Group * (Age.z + Sex) + Trial.z +
(1 + Trial.z || AnimalID), family = binomial

Conditional model – Summary

	Estimate	Std.Error	z-value	Pr(>|z|)	

(Intercept)	-2.80	0.59	-4.78	0.00	***
GroupExperimental	0.54	0.65	0.83	0.41	
Age.z	-0.23	0.29	-0.80	0.43	
SexM	0.37	0.76	0.49	0.63	
Trial.z	-0.06	0.20	-0.32	0.75	
GroupExperimental:Age.z	1.07	0.44	2.43	0.02	*
GroupExperimental:SexM	-0.16	0.93	-0.17	0.86	

Null model comparison

	npar	AIC	BIC	logLik	deviance	Chisq	Df	Pr(>Chisq)
null.Sniff2.I	5.00	484.87	506.94	-237.44	474.87			
full.Sniff2.I	10.00	486.09	530.23	-233.04	466.09	8.78	5.00	0.12

Confidence intervals

	LOWER CI (2.5%)	UPPER CI (97.5%)
(Intercept)	-3.95	-1.65
GroupExperimental	-0.74	1.82
Age.z	-0.81	0.34
SexM	-1.12	1.86
Trial.z	-0.46	0.33
GroupExperimental:Age.z	0.21	1.93
GroupExperimental:SexM	-1.99	1.66

6.3.2 LOOKING AT EXPERIMENTER

Formula: Looking.at.experimenter.GT.Prop_Sum ~ Group * (Age.z + Sex) + Trial.z +
(1 + Trial.z || AnimalID), family = beta

Conditional model – Summary

	Estimate	Std.Error	t-value
(Intercept)	0.48	0.04	12.91
GroupExperimental	-0.05	0.04	-1.05
Age.z	0.01	0.02	0.29
SexM	-0.03	0.05	-0.54

Trial.z	-0.01	0.01	-0.55
GroupExperimental:Age.z	0.00	0.03	0.01
GroupExperimental:Sex	0.03	0.07	0.51

Null model comparison

	npar	AIC	BIC	logLik	deviance	Chisq	Df	Pr(>Chisq)
null.LookEPG.I	5.0	-337.1	-315.0	173.5	-347.1			
full.LookEPG.I	10.0	-328.5	-284.4	174.3	-348.5	1.5	5.0	0.9

Confidence intervals

	LOWER CI (2.5%)	UPPER CI (97.5%)
(Intercept)	0.41	0.55
GroupExperimental	-0.13	0.04
Age.z	-0.03	0.05
SexM	-0.13	0.07
Trial.z	-0.03	0.01
GroupExperimental:Age.z	-0.06	0.06
GroupExperimental:SexM	-0.09	0.16

6.3.3 PROXIMITY TO EXPERIMENTER

Formula: Proximity.experimenter.Prop_Sum ~ Group * (Age.z + Sex) + Trial.z +
(1 + Trial.z || AnimalID), family = beta

Conditional model – Summary

	Estimate	Std.Error	z-value	Pr(>|z|)	
(Intercept)	-2.01	0.09	-22.50	<2e-16	***
GroupExperimental	0.22	0.09	2.36	0.02	*
Age.z	-0.02	0.04	-0.43	0.67	
SexM	0.04	0.11	0.40	0.69	
Trial.z	0.03	0.03	0.97	0.33	
GroupExperimental:Age.z	0.06	0.06	0.96	0.34	
GroupExperimental:SexM	-0.10	0.14	-0.76	0.45	

Null model comparison

	Df	AIC	BIC	logLik	deviance	Chisq	Df	Pr(>Chisq)	
null.ProxEP.I	5	-8441.7	-8416.3	4225.9	-8451.7				
red.ProxEP.I	8	-8442.5	-8401.8	4229.3	-8458.5	6.7727	3	0.07951	.

Reduced model – summary

	Estimate	Std.Error	z-value	Pr(>|z|)	
(Intercept)	-2.01	0.09	-22.50	<2e-16	***
GroupExperimental	0.17	0.068	2.509	0.0121	*
Age.z	0.008	0.032	0.264	0.7914	
SexM	-0.02	0.066	-0.323	0.7469	
Trial.z	0.029	0.031	0.937	0.3488	

Confidence intervals

	Lower CI (2.5%)	Upper CI (97.5%)
(Intercept)	-2.13	-1.82
GroupExperimental	0.04	0.31
Age.z	-0.05	0.07
SexM	-0.15	0.11
Trial.z	-0.03	0.09

Posthoc (Group)

Group	estimate	SE	df	z-ratio	p-value
Control - Experimental	-0.172	0.0687	Inf	-2.509	0.01

6.3.4 TAIL HIGH

Formula: Tail.wagging.high.Prop_sum ~ Group * (Age.z + Sex) + Trial.z +

(1 + Trial.z || AnimalID), family = beta

Conditional model – Summary

	Estimate	Std.Error	z-value	Pr(>|z|)	
(Intercept)	1.88	0.28	6.59	0.00	***
GroupExperimental	-0.63	0.32	-1.96	0.05	.

Age.z	0.03	0.15	0.20	0.84	
SexM	-0.62	0.39	-1.60	0.11	
Trial.z	-0.25	0.06	-4.00	0.00	***
GroupExperimental:Age.z	-0.11	0.22	-0.47	0.64	
GroupExperimental:SexM	0.87	0.48	1.81	0.07	.

Null model comparison

	Df	AIC	BIC	logLik	deviance	Chisq	Df	Pr(>Chisq)
null.TailHP.I	5	-3752.20	-3730.10	1881.10	-3762.20			
full.TailHP.I	10	-3746.60	-3702.40	1883.30	-3766.60	4.38	5.00	0.50

Confidence intervals

	LOWER CI (2.5%)	UPPER CI (97.5%)
(Intercept)	1.32	2.44
GroupExperimental	-1.27	0.00
Age.z	-0.26	0.32
SexM	-1.38	0.14
Trial.z	-0.38	-0.13
GroupExperimental:Age.z	-0.54	0.33
GroupExperimental:SexM	-0.07	1.81

6.3.5 TAIL LOW

Formula: Tail.wagging.low.~ Group * (Age.z + Sex) + Trial.z +

(1 + Trial.z || AnimalID), family = binomial

Conditional model – Summary

	Estimate	Std.Error	z-value	Pr(>|z|)	
(Intercept)	-28.31	96541.83	0.00	1.00	
GroupExperimental	1.79	0.87	2.06	0.04	*
Age.z	0.08	0.36	0.21	0.83	
SexM	-0.06	0.74	-0.08	0.93	
Trial.z	0.25	0.14	1.85	0.07	.

GroupExperimental:Age.z	-0.31	0.69	-0.45	0.65	
GroupExperimental:SexM	-25.07	96541.83	0.00	1.00	

Null model comparison

	npar	AIC	BIC	logLik	deviance	Chisq	Df	Pr(>Chisq)	
null.TailLP2.I	4	471.5	491.9	-231.7	463.5				
full.TailLP2.I	9	469.1	515.1	-225.6	451.1	12.4	5.0	0.0	*

Reduced model – Summary

	Estimate	Std.Error	z-value	Pr(>|z|)	
(Intercept)	-28.31	96541.83	0.00	1.00	
GroupExperimental	1.79	0.87	2.06	0.04	*
Age.z	0.08	0.36	0.21	0.83	
SexM	-0.06	0.74	-0.08	0.93	
Trial.z	0.25	0.14	1.85	0.07	.

Confidence intervals

	LOWER CI (2.5%)	UPPER CI (97.5%)
(Intercept)	-7.54	-3.68
GroupExperimental	0.09	3.48
Age.z	-0.63	0.79
SexM	-1.51	1.38
Trial.z	-0.02	0.51

Post-hoc (Group)

Group	estimate	SE	df	z-ratio	p-value
Control	-1.79	0.87	Inf	-2.06	0.04

6.3.6 TRIAL NOT ACCOMPLISHED

Formula: Tail.wagging.low.~ Group * (Age.z + Sex) + Trial.z +

(1 + Trial.z || AnimalID), family = binomial

Conditional model – Summary

	Estimate	Std.Error	z-value	Pr(>|z|)	
(Intercept)	-2.15	0.97	-2.21	0.03	*
GroupExperimental	0.57	0.97	0.59	0.55	
Age.z	-0.21	0.46	-0.46	0.64	
SexM	0.21	1.17	0.18	0.86	
GroupExperimental:Age.z	0.75	0.68	1.10	0.27	
GroupExperimental:SexM	-0.21	1.41	-0.15	0.88	

Null model comparison

	npar	AIC	BIC	logLik	deviance	Chisq	Df	Pr(>Chisq)
null.NotAcc.I	2	128.36	132.58	-62.18	124.36			
full.NotAcc.I	7	136.37	151.14	-61.184	122.37	1.9931	5	0.85

Confidence intervals

	LOWER CI (2.5%)	UPPER CI (97.5%)
(Intercept)	-1.97	-1.96
GroupExperimental	0.40	0.40
Age.z	0.14	0.15
SexM	0.02	0.02

6.3.7 SESSION TERMINATED

Fisher's Exact Test for Count Data

p-value = 0.08092

alternative hypothesis: true odds ratio is not equal to 1

95 percent confidence interval: 0.7665582 ; 288.6528125

sample estimates, odds ratio: 6.186259
